# Antioxidant, Redox, and Immunomodulatory Effects of *Hypericum perforatum* in the *Galleria mellonella*: A 3R-Oriented Invertebrate Model

**DOI:** 10.3390/biomedicines14061297

**Published:** 2026-06-07

**Authors:** Fatih Battal, Serhat Kaya, Hasan Ali Kiraz

**Affiliations:** 1 Department of Pediatrics, Faculty of Medicine, Çanakkale Onsekiz Mart University, Çanakkale 17100, Türkiye; fatihbattal@comu.edu.tr; 2 Department of Biology, Faculty of Science, Çanakkale Onsekiz Mart University, Çanakkale 17100, Türkiye; 3 Department of Anesthesiology and Reanimation, Faculty of Medicine, Çanakkale Onsekiz Mart University, Çanakkale 17100, Türkiye; hakiraz@comu.edu.tr

**Keywords:** antioxidants, hemolymph, larva, oxidative stress, plant extracts, *Hypericum perforatum*, *Galleria mellonella*

## Abstract

**Background/Objectives**: *Hypericum perforatum* L. (St. John’s Wort) is extensively utilized in ethnopharmacology due to its anti-inflammatory and immunomodulatory properties. However, its effects on the interaction between innate immunity and oxidative homeostasis remain incompletely characterized, particularly in alternative invertebrate models. This study aimed to evaluate the effects of *H. perforatum* extract on oxidative homeostasis and protein metabolism using the *Galleria mellonella* model, a 3R-compliant and ethically sustainable platform for preliminary immunological and redox-related screening. **Methods**: Last instar *G. mellonella* larvae were administered increasing concentrations of *H. perforatum* extract (0.001–20 mg mL^−1^) by intrahemocoelic injection. After 24 h, hemolymph samples were analyzed for total protein (TP), total hemocyte count (THC), encapsulation and melanization responses, superoxide dismutase (SOD) activity, catalase (CAT) activity, and malondialdehyde (MDA) levels. The phytochemical profile of the extract was additionally evaluated using GC–MS analysis. **Results**: Significant group-dependent alterations were observed in TP levels and THC values, with the HP-2 group demonstrating the highest hemocyte counts and enhanced strong encapsulation responses. Higher extract concentrations, particularly HP-4, were associated with increased weak encapsulation profiles, suggesting altered cellular immune organization. Melanization responses became significantly elevated at 24 h following treatment. In contrast, SOD activity, CAT activity, and MDA levels did not differ significantly among groups, indicating preservation of oxidative homeostasis under the tested conditions. **Conclusions**: *H. perforatum* extract induced dose-dependent modulation of cellular and humoral immune responses in *G. mellonella* without evidence of detectable oxidative disruption during acute exposure. These findings support the utility of the *G. mellonella* model for preliminary evaluation of botanical immunomodulators and suggest that *H. perforatum* may influence immunophysiological pathways independently of overt oxidative toxicity.

## 1. Introduction

*Hypericum perforatum* L., commonly known as St. John’s Wort, is one of the most extensively studied medicinal plants in ethnopharmacology, owing to its broad spectrum of biological activities, including anti-inflammatory, antimicrobial, wound-healing, and neuroprotective effects [1]. These therapeutic properties are largely attributed to its diverse phytochemical composition, particularly hypericin, hyperforin, flavonoids, and phenolic antioxidants, which collectively modulate inflammatory signaling pathways and cellular redox balance [2]. Although numerous mammalian studies have demonstrated its efficacy in tissue repair and neuroimmune regulation [3,4], the systemic interaction between its immunomodulatory effects and oxidative homeostasis remains incompletely characterized, especially within developmental biological models.

In recent years, increasing ethical and regulatory concerns in experimental research have accelerated interest in alternative model organisms compatible with the “3R” principles (Replacement, Reduction, Refinement). Among these, the insect model *Galleria mellonella* has gained prominence due to its evolutionarily conserved innate immune mechanisms, including phagocytic hemocytes, antimicrobial peptide production, and melanization responses that functionally parallel vertebrate inflammatory pathways [5,6]. These characteristics make the model particularly suitable for studying host–response dynamics and systemic physiological responses while minimizing ethical burden.

The insect immune system represents a highly organized defense network based on the coordinated interaction of physical barriers, cellular defense mechanisms, and humoral factors. Although insects were traditionally considered to possess only innate immunity, recent studies have demonstrated the existence of memory-like responses, including immune priming and transgenerational immune transfer [7,8,9,10]. In insects, immune responses are mediated not only by physical barriers such as the cuticle and peritrophic membrane but also by hemocyte-mediated cellular reactions and humoral components, including antimicrobial peptides, lysozyme, reactive oxygen species (ROS), and the prophenoloxidase (proPO) system [11]. Recognition of pathogen-associated molecular patterns (PAMPs) by pattern recognition receptors (PRRs) activates the Toll, IMD, and proPO pathways, ultimately triggering defense reactions such as phagocytosis, nodulation, encapsulation, and melanization [7,12].

Activation of innate immunity is closely associated with increased metabolic demand and ROS production [13]. In particular, ROS generated during respiratory burst and melanization processes play critical roles in pathogen elimination; however, excessive accumulation may lead to lipid peroxidation, protein oxidation, and cellular membrane damage [14]. Antioxidant enzymes such as superoxide dismutase (SOD) and catalase (CAT) constitute major defense systems against oxidative injury, whereas malondialdehyde (MDA) is widely used as a biomarker of lipid peroxidation [15]. Therefore, evaluation of the relationship between immune activation and oxidative stress is considered an important approach for understanding the biological effects of plant-derived compounds.

Total hemocyte count (THC) is one of the most frequently used physiological parameters for evaluating immune capacity in insects. Hemocytes are directly involved in major defense reactions, including phagocytosis, nodulation, encapsulation, wound repair, and melanin formation [7]. In addition to their role in cellular immunity, hemocytes also play critical roles in the synthesis of antimicrobial peptides and various humoral defense proteins [16,17]. Alterations in THC levels are therefore considered important indicators of immune responses induced by infection, toxic exposure, or environmental stress conditions [16,18].

Encapsulation is one of the principal cellular defense mechanisms responsible for eliminating foreign bodies too large to be removed by phagocytosis [19]. During this process, hemocytes form multilayered cellular aggregates around invading structures, thereby physically isolating the foreign material [20,21]. Recent studies have demonstrated that encapsulation is not merely passive cellular accumulation but rather an active defense response involving complex signaling and immune regulation [22]. Furthermore, certain parasitoids have been reported to suppress encapsulation through polydnaviruses or immunosuppressive factors [23].

Melanization represents one of the fastest and most effective humoral defense mechanisms in insects and commonly occurs in association with encapsulation responses [24]. This process begins with the conversion of prophenoloxidase into active phenoloxidase through a serine protease cascade, ultimately resulting in melanin synthesis [25]. The resulting melanin layer immobilizes invading organisms, while quinones and ROS generated during the process exert direct cytotoxic effects [26]. However, excessive melanization may also induce oxidative injury in host tissues. Consequently, inhibitory molecules such as serpins play critical roles in maintaining the balance between immune defense and tissue integrity [27,28].

Previous studies have demonstrated that *H. perforatum* extracts may exert immunostimulatory effects in invertebrate models and modulate cellular immune responses. However, the incomplete characterization of the relationship between its chemical antioxidant capacity and systemic antioxidant responses in vivo suggests that this plant may exert its effects primarily through metabolic adaptation processes rather than direct radical scavenging activity alone. Moreover, despite the increasing use of herbal preparations as complementary therapeutic agents, systematic preclinical data regarding their physiological and oxidative stress-related effects remain limited [29,30,31,32,33,34].

Accordingly, the present study aimed to investigate the effects of *H. perforatum* extract on oxidative stress parameters, protein metabolism, and immunological response indicators in *G. mellonella* larvae. For this purpose, SOD and CAT activities, MDA levels, total protein profiles, total hemocyte counts, encapsulation responses, and melanization processes were evaluated together to characterize the physiological alterations induced by acute extract exposure. The primary hypothesis of the study was that *H. perforatum* extract may modulate immunological and metabolic responses without inducing marked lipid peroxidation or overt oxidative injury.

## 2. Materials and Methods

### 2.1. Experimental Model and Insect Rearing

Last instar larvae of the greater wax moth, *G. mellonella*, were obtained from a synchronized laboratory colony maintained at the Insect Physiology Laboratory, Department of Biology, Çanakkale Onsekiz Mart University. To establish a homogeneous experimental cohort, adult moths (4 females and 2 males) were placed in 1 L glass containers supplied with natural dark honeycomb as an oviposition substrate. Colonies were maintained under controlled environmental conditions (30 ± 1 °C, 65 ± 5% relative humidity, constant darkness). Larvae were fed ad libitum with a semi-synthetic diet composed of dark honeycomb, wheat bran, honey, distilled water, and glycerol. Only healthy, cream-colored final instar larvae weighing 0.18 ± 0.02 g were selected to minimize physiological variability. Biochemical measurements were performed by an investigator blinded to group allocation until completion of statistical analysis.

### 2.2. Plant Material and Extraction Procedure

Aerial parts (flowers and leaves) of *H. perforatum* were collected from Yağcılar, Çanakkale, Türkiye (35T 453758.424 4437824.336), and taxonomically authenticated by a plant taxonomist. The plant material was shade-dried at room temperature in a ventilated environment. Dried material was subjected to Soxhlet extraction using 70% ethanol for exhaustive recovery of bioactive compounds. The solvent was removed under reduced pressure at 35 °C using a rotary evaporator. The extraction yield was determined as 7.2% (*w*/*w*) based on the initial dry weight of the plant material.

Stock solutions were prepared in sterile distilled water to obtain the following concentrations: 0.001 mg mL^−1^ (HP-1), 0.0025 mg mL^−1^ (HP-2), 2.5 mg mL^−1^ (HP-3), and 20 mg mL^−1^ (HP-4). The doses used in this study were selected based on the preliminary screening reported by Genç et al. [16], which identified 0.001–0.0025 mg mL^−1^ as the lowest concentrations capable of significantly modulating phenoloxidase (PO) activity, while responses at 20 mg mL^−1^ suggested a high-dose activity region. Therefore, 2.5 mg mL^−1^ was included as an intermediate concentration to evaluate the transition between low- and high-dose responses. All selected doses were substantially below the maximum tolerated concentration (200 mg mL^−1^), and were considered non-lethal for evaluating immunometabolic responses.

### 2.3. Experimental Design and Microinjection

Larvae were randomly assigned to experimental groups (*n* = 16 per group). Prior to injection, larvae were immobilized on ice to minimize stress-induced immune activation. A fixed volume (5 µL) of extract solution was administered by intrahemocoelic injection into the last right proleg using a calibrated glass microsyringe under stereomicroscopic guidance.

Two control groups were included: an untreated control and a sham control (sterile distilled water). No statistically significant differences were observed between untreated and sham-injected groups, indicating that the injection procedure itself did not induce detectable oxidative alterations.

Following the injection, larvae were incubated for 24 h. The 24 h sampling point was selected based on previous findings indicating that immune-related gene expression and protein modulation in *G. mellonella* typically reach peak levels within this timeframe [16]. Additionally, this duration was maintained to ensure consistency with our preliminary transcriptomic data and to avoid the physiological interference of pupation-related protein redistribution.

### 2.4. Hemolymph Collection and Processing

Hemolymph was collected by puncturing the cuticle anterior to the prolegs using a sterile needle. Approximately 30 µL of hemolymph from each larva was immediately diluted in 270 µL cold phosphate-buffered saline (PBS) to prevent melanization. Samples were centrifuged at 10,000× *g* for 5 min at 4 °C to obtain cell-free supernatant. The resulting plasma fraction was rapidly frozen in liquid nitrogen and stored at −80 °C until biochemical analysis.

### 2.5. Biochemical Assays

All measurements were performed using four independent biological replicates, with four larvae per experimental group in each replicate (total *n* = 16 per group). Absorbance readings were conducted using a Multiskan GO microplate spectrophotometer (Thermo Scientific, Vantaa, Finland).

#### 2.5.1. Total Protein

Total protein concentration was determined using the Bradford assay. A 5 µL aliquot of the hemolymph–phosphate buffer mixture was added to each well of a 96-well microplate, followed by 155 µL distilled water and 40 µL Bradford reagent. The plate was incubated for 30 min at room temperature, and absorbance was measured at 595 nm.

#### 2.5.2. Superoxide Dismutase (SOD) Activity

SOD activity was determined based on the inhibition of nitroblue tetrazolium (NBT) reduction. A 6.5 µL sample of the hemolymph–phosphate buffer mixture was mixed with 190 µL SOD reagent and 3.5 µL xanthine oxidase. The reaction mixture was incubated for 20 min under light exposure at room temperature. Subsequently, 6.5 µL CuCl_2_ (0.8 mM) was added, and absorbance was measured at 560 nm.

#### 2.5.3. Catalase (CAT) Activity

Catalase activity was measured by monitoring the decomposition rate of hydrogen peroxide. A 6.5 µL sample of hemolymph–buffer mixture was mixed with 60 µL phosphate buffer (50 mM, pH 7.2) and 133.5 µL H_2_O_2_ (30 mM). Kinetic absorbance readings were recorded at 240 nm at 10 s intervals for 2 min.

#### 2.5.4. Lipid Peroxidation (MDA)

Malondialdehyde (MDA) levels were quantified using the thiobarbituric acid reactive substances (TBARS) assay. A 75 µL sample of hemolymph–buffer mixture was mixed with 150 µL TBA–TCA reagent and incubated at 90 °C for 20 min. After cooling, absorbance was measured at 532 nm.

### 2.6. Total Hemocyte Count (THC)

Total hemocyte count (THC) was determined according to the method described by Kaya et al. [35]. At 24 h post-injection, a small puncture was created in the segment immediately anterior to the prolegs using a sterile injection needle. Subsequently, 4 µL hemolymph was collected and immediately transferred into a microcentrifuge tube containing 36 µL anticoagulant solution (0.098 M NaOH, 0.186 M NaCl, 0.017 M Na_2_EDTA and 0.041 M Citric acid, pH: 4.5).

A 10 µL aliquot of the resulting hemolymph suspension was loaded onto an improved Neubauer hemocytometer (Superior, Morris, MN, USA), and hemocyte counts were performed under a phase-contrast microscope (Olympus BX-51, Olympus Optical Co. Ltd., Tokyo, Japan). THC values were expressed as the number of hemocytes per milliliter of hemolymph.

### 2.7. Encapsulation and Melanization Responses

Encapsulation and melanization responses were evaluated as indicators of functional innate immune activity. At 24 h post-injection, larvae from control and treatment groups were injected with 10 µL phosphate-buffered saline (PBS) containing approximately 15–20 Sephadex A-25 chromatography beads (Sigma-Aldrich, Darmstadt, Germany). To facilitate visualization within the hemocoel, beads were pre-stained with 1% Brilliant Blue G (Sigma-Aldrich, Darmstadt, Germany).

At 4 and 24 h following bead injection, larvae were dissected under a stereomicroscope (Leica EZ4, Leica Microsystems, Kista, Sweden), and the recovered beads were examined under a phase-contrast microscope. Encapsulation intensity was classified according to the criteria previously described by Kaya et al. [35], based on the number of hemocyte layers surrounding each bead. Simultaneously, the presence or absence of melanization around the beads was also recorded.

### 2.8. GC–MS Analysis

The phytochemical profiling of the *H. perforatum* extract was performed using a Trace 1300 Gas Chromatograph coupled with an ISQ 7000 Mass Spectrometer (Thermo Scientific, Waltham, MA, USA). Samples were introduced via a TriPlus RSH Autosampler (Thermo Scientific, Waltham, MA, USA) to ensure high injection precision. Separation was carried out on a TG-5MS capillary column (30 m × 0.25 mm i.d., 0.25 μm film thickness; Thermo Scientific). Helium was used as the carrier gas at a constant flow rate of 1.0 mL min^−1^. The total chromatographic run time was 57.11 min. The MS transfer line and ion source temperatures were set at 280 °C and 230 °C, respectively. Mass spectra were acquired in electron impact (EI) mode at 70 eV, with a mass scanning range of 50–500 *m*/*z*. Compound identification was based on a comparison of mass spectra with the NIST and Wiley libraries, alongside retention indices. The present GC–MS analysis should be interpreted as a preliminary qualitative phytochemical screening rather than a definitive quantitative characterization.

### 2.9. Statistical Analysis

All statistical analyses were performed using IBM SPSS Statistics version 20.0 (IBM Corp., Armonk, NY, USA). Data are presented as mean ± standard deviation (SD). The normality of data distribution and homogeneity of variances were evaluated using the Shapiro–Wilk and Levene tests, respectively.

Comparisons among experimental groups were performed using one-way analysis of variance (ANOVA). When significant overall differences were detected, Tukey’s Honestly Significant Difference (HSD) test was used for post hoc pairwise comparisons. A *p*-value < 0.05 was considered statistically significant. All statistical results are presented in Supplementary Material S2.

## 3. Results

### 3.1. Gas Chromatography–Mass Spectrometry (GC–MS) Profiling of the Extract

GC–MS analysis was performed to obtain a qualitative profile of the *H. perforatum* extract. Profiling revealed the presence of phenolic derivatives, fatty acid esters, and minor heterocyclic compounds consistent with the known phytochemical composition of *H. perforatum*. Siloxane-related peaks detected at early retention times were considered instrumental artifacts and were excluded from biological interpretation.

The tentatively identified major compounds are presented in Table 1. The complete GC–MS chromatogram and full list of tentatively identified compounds are provided in the Supplementary Material (Supplementary Material S1). The detection of these bioactive constituents, particularly phenolic and fatty acid derivatives, provides a supportive preliminary phytochemical context, as these classes of compounds are known to influence immune signaling and redox homeostasis in insect models. The detection of these putative constituents provides a supportive preliminary phytochemical context for the observed physiological responses; however, direct compound-specific attribution requires targeted quantitative analyses.

### 3.2. Total Protein Levels

Hemolymph total protein (TP) concentrations differed significantly among experimental groups (one-way ANOVA, F(5,90) = 4.764, *p* < 0.001; η^2^ = 0.209, 95% CI [0.046, 0.313]). Post hoc analyses demonstrated that this effect was primarily driven by a significant reduction in the HP-2 group (0.4026 absorbance units) compared with the untreated control group (0.4497 absorbance units) (*p* = 0.036).

**Figure 1:**
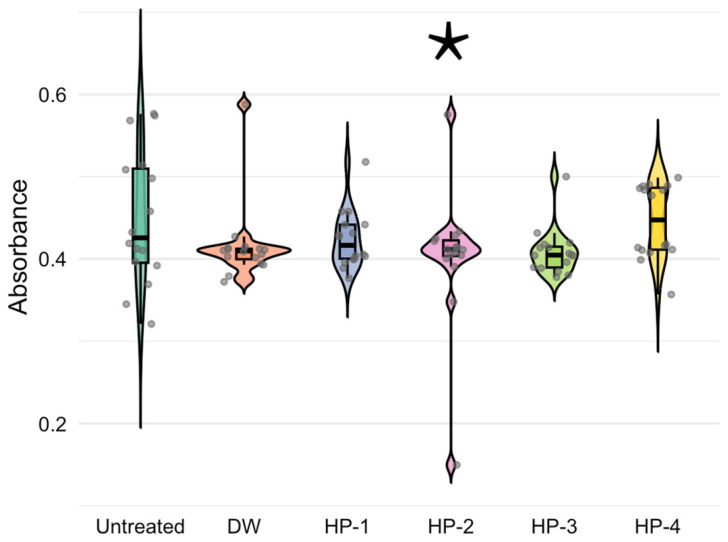
Effect of *H. perforatum* injection on total protein levels in *G. mellonella* hemolymph. Distribution of hemolymph total protein (TP) concentrations across experimental groups at 24 h post-injection. Box-and-whisker plots represent the median, interquartile range (IQR), and minimum–maximum values. Asterisks (*) indicate statistically significant differences between the groups. A significant reduction in TP concentration was observed in the HP-2 group (0.0025) compared with the untreated control group (*p* = 0.036). Statistical analyses were performed using one-way ANOVA followed by Tukey’s HSD post hoc test (F(5,90) = 4.764, *p* < 0.001; η^2^ = 0.209, 95% CI [0.046, 0.313]). (Untreated: control; DW: Distilled water/Sham control; HP-1 to HP-4: Graded doses of extract).

As illustrated in Figure 1, TP values exhibited a non-linear dose–response distribution, with the lowest median values observed in the HP-2 group. Although higher extract concentrations did not induce further reductions in protein levels, the selective decrease observed at the intermediate–low concentration suggests a dose-dependent modulation of hemolymph protein metabolism rather than generalized protein depletion.

### 3.3. Catalase Activity

Catalase (CAT) activity remained stable across all experimental groups, and no statistically significant differences were observed following extract administration (F(5,90) = 0.254, *p* = 0.939; η^2^ = 0.0139, 95% CI [0.000, 0.023]).

**Figure 2:**
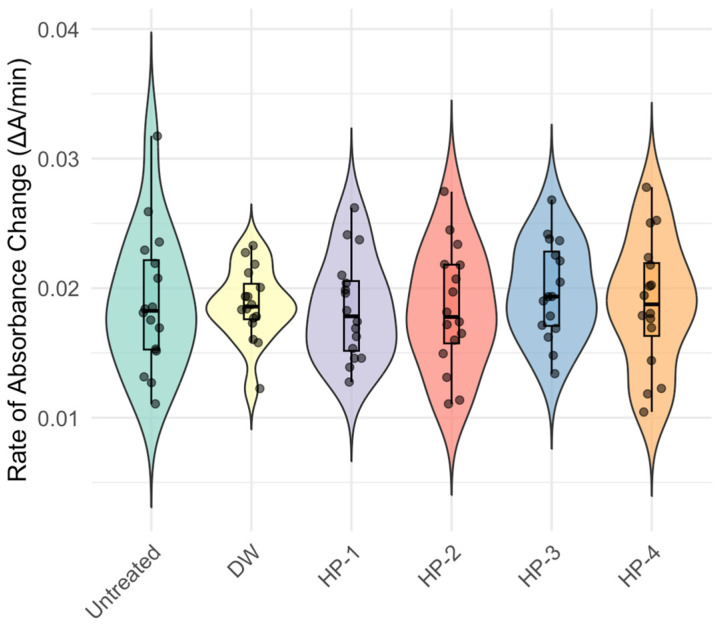
Effect of *H. perforatum* injection on catalase (CAT) activity in *G. mellonella* hemolymph. Violin-box plots illustrating catalase (CAT) activity levels across experimental groups at 24 h post-injection. No statistically significant differences were detected among control and extract-treated groups (F(5,90) = 0.254, *p* = 0.939; η^2^ = 0.0139, 95% CI [0.000, 0.023]). Data are presented as mean ± SD of four independent replicates. (Untreated: control; DW: Distilled water/Sham control; HP-1 to HP-4: Graded doses of extract).

As shown in Figure 2, CAT distributions demonstrated substantial overlap between groups, with comparable median values and interquartile ranges. These findings indicate that *H. perforatum* exposure did not produce a measurable alteration in hydrogen peroxide detoxification capacity under the experimental conditions used in this study.

### 3.4. Superoxide Dismutase Activity

No significant differences were detected in superoxide dismutase (SOD) activity among the treatment groups (F(5,90) = 0.491, *p* = 0.782; η^2^ = 0.027, 95% CI [0.000, 0.061]).

**Figure 3:**
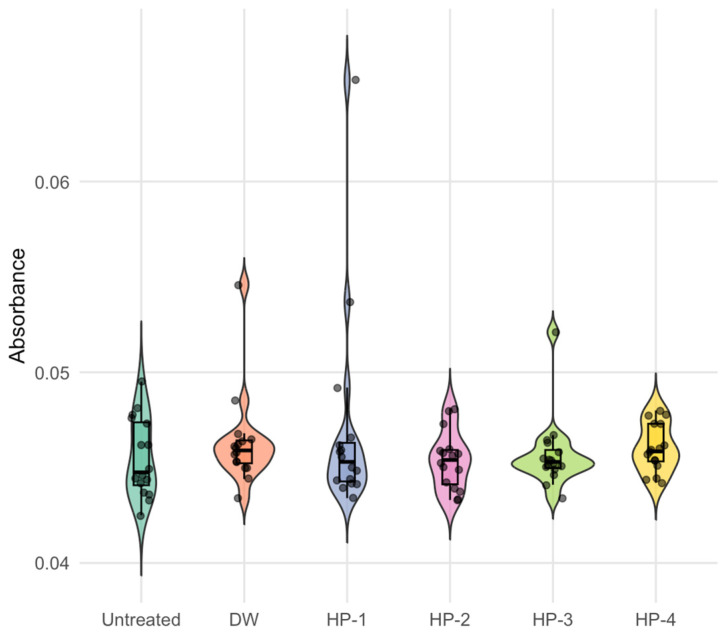
Effect of *H. perforatum* injection on superoxide dismutase (SOD) activity in *G. mellonella* hemolymph. Graphical representation of superoxide dismutase (SOD) activity levels in *G. mellonella* larvae at 24 h post-injection following administration of increasing concentrations of *H. perforatum* extract. SOD activity values demonstrated minimal dispersion and substantial overlap among experimental groups, with no statistically significant differences detected between treatment and control groups (F(5,90) = 0.491, *p* = 0.782; η^2^ = 0.027, 95% CI [0.000, 0.061]). (Untreated: control; DW: Distilled water; HP-1 to HP-4: Graded doses of *H. perforatum* extract).

As presented in Figure 3, SOD values clustered tightly with minimal dispersion across all groups. The preservation of baseline SOD activity suggests that acute extract exposure did not induce substantial enzymatic antioxidant activation or suppression during the 24 h observation period.

### 3.5. Lipid Peroxidation (MDA)

**Figure 4:**
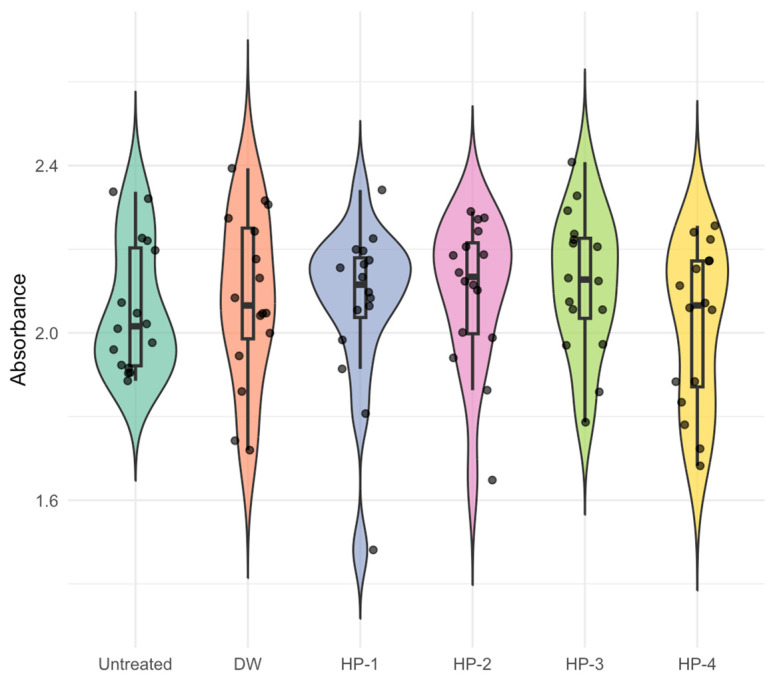
Effect of *H. perforatum* extract on malondialdehyde (MDA) levels in *G. mellonella* hemolymph. Malondialdehyde (MDA) concentrations, used as an indicator of lipid peroxidation, were measured in hemolymph samples at 24 h post-injection. Distribution patterns demonstrated comparable central tendencies and overlapping variability across all experimental groups, with no statistically significant differences detected following extract exposure (F(5,90) = 1.333, *p* = 0.258; η^2^ = 0.069, 95% CI [0.000, 0.139]). (Untreated: control; DW: Distilled water; HP-1 to HP-4: Graded doses of extract).

Malondialdehyde (MDA) concentrations did not differ significantly among experimental groups (F(5,90) = 1.333, *p* = 0.258; η^2^ = 0.069, 95% CI [0.000, 0.139]). The distributions shown in Figure 4 demonstrated similar central tendencies and overlapping variability patterns across all treatments. Collectively, these findings suggest that the administered extract concentrations did not induce detectable lipid peroxidation or oxidative membrane damage within the investigated timeframe.

### 3.6. Total Hemocyte Count (THC)

**Figure 5:**
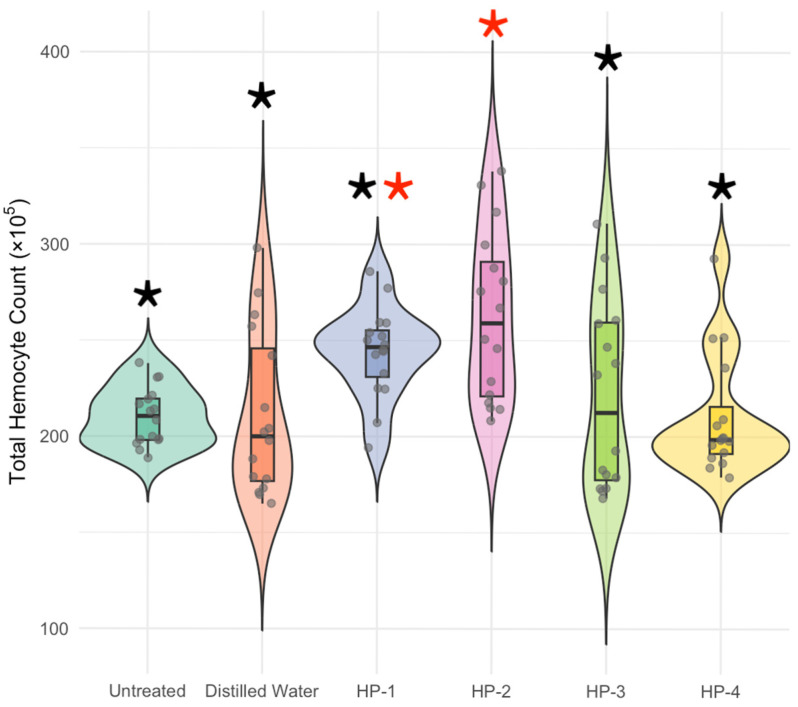
Effect of *H. perforatum* extract on total hemocyte count (THC) in *G. mellonella* larvae. Violin-box plots illustrating total hemocyte count (THC) levels in *G. mellonella* larvae at 24 h following administration of increasing concentrations of *H. perforatum* extract. Each group represents 16 biological replicates. Box plots indicate the median and interquartile range, while violin distributions illustrate data density and variability within groups. Groups sharing identical asterisk colors did not differ significantly from each other, whereas groups marked with different colored asterisks exhibited statistically significant differences.

The effects of *H. perforatum* extract on total hemocyte count are presented in Figure 5. Statistical analysis demonstrated a significant and large overall group effect on THC levels (one-way ANOVA, F(5,90) = 5.36, *p* = 0.00023; η^2^ = 0.23, 95% CI [0.061, 0.334]).

The highest mean hemocyte count was observed in the HP-2 group (263.75 × 10^5^ cells), whereas the lowest mean values were detected in the untreated (208.19 × 10^5^ cells) and HP-4 groups (208.56 × 10^5^ cells). Post hoc analyses revealed no significant differences among the untreated, DW, HP-3, and HP-4 groups. However, the HP-2 group demonstrated significantly higher THC values compared with these groups. Although the difference between HP-1 and untreated controls was only marginally significant, no additional pairwise differences involving HP-1 reached statistical significance.

Overall, these findings indicate that intermediate–low concentrations of *H. perforatum* selectively enhanced circulating hemocyte abundance, whereas higher extract concentrations did not further augment cellular immune activation.

### 3.7. Encapsulation Responses

At the 4 h assessment, no statistically significant differences were detected among groups regarding the proportion of non-encapsulated (“None”) beads (ANOVA *p* = 0.088; Kruskal–Wallis *p* = 0.204). Descriptive analyses supported these findings, with low and comparable None percentages across all groups (Table 2).

In contrast, significant group-dependent differences were observed for both weak and strong encapsulation responses. For weak encapsulation, both ANOVA (*p* = 0.0025; η^2^ = 0.182) and Kruskal–Wallis analyses (*p* = 0.006) demonstrated significant differences among groups. Tukey post hoc analysis revealed that the HP-3 group exhibited significantly higher weak encapsulation responses than both the DW and HP-2 groups (*p* < 0.05). Descriptive analyses demonstrated that the HP-3 group showed the highest weak encapsulation rate (85.0%), whereas the HP-2 group exhibited the lowest proportion (64.1%) (Table 2).

Similarly, strong encapsulation responses differed significantly among groups (ANOVA *p* = 0.000285; η^2^ = 0.226; Kruskal–Wallis *p* = 0.0025). Post hoc comparisons demonstrated that the HP-2 group had significantly higher strong encapsulation values compared with both the HP-3 and HP-4 groups (*p* < 0.01). No additional pairwise differences reached statistical significance. Descriptive analyses showed that strong encapsulation was highest in the HP-2 group (34.1%) and lowest in the HP-3 group (11.2%).

At 24 h, the proportion of non-encapsulated beads remained statistically comparable among groups (Kruskal–Wallis *p* = 0.115), with consistently low None percentages observed across all experimental conditions (Table 2).

In contrast, both weak and strong encapsulation responses demonstrated highly significant intergroup differences (Kruskal–Wallis for both variables, *p* < 0.001; ε^2^ = 0.44 and 0.41, respectively, indicating large effect sizes). Dunn post hoc analyses with Bonferroni correction revealed that the HP-4 group exhibited significantly higher weak encapsulation values than untreated, DW, HP-1, and HP-2 groups (all *p* < 0.001). Additionally, the HP-3 group showed higher weak encapsulation values compared with HP-1 (*p* = 0.003).

Descriptive analyses demonstrated that weak encapsulation responses reached the highest levels in the HP-4 group (66.8%), whereas untreated, DW, HP-1, and HP-2 groups showed lower and comparable values (Table 2).

Strong encapsulation responses also differed significantly among groups. The HP-4 group exhibited significantly lower strong encapsulation values than the DW, HP-1, HP-2, and untreated groups (all *p* < 0.02). Furthermore, HP-1 demonstrated significantly higher strong encapsulation responses compared with HP-3 (*p* = 0.003). Descriptive statistics showed that strong encapsulation rates were highest in the HP-1 group (59.8%) and lowest in the HP-4 group (31.8%) (Table 2).

### 3.8. Melanization Responses

At the 4 h time point, no statistically significant differences were observed among groups regarding either non-melanized (None) or melanized bead proportions (Kruskal–Wallis *p* = 0.172 for both variables; ε^2^ = 0.030, indicating a small effect size). Because of deviations from normality assumptions, Kruskal–Wallis results were considered primary; however, ANOVA analyses similarly confirmed the absence of significant intergroup differences (all *p* > 0.05) (Table 3).

Descriptive analyses demonstrated relatively comparable melanization distributions among groups. Although the HP-2 group exhibited a numerically higher melanized bead proportion (81.3%), this increase did not reach statistical significance. Collectively, these findings suggest that extract administration did not substantially alter early melanization responses during the initial 4 h period (Table 3).

At 24 h, significant differences emerged among experimental groups. For the non-melanized (None) variable, Kruskal–Wallis analysis demonstrated a statistically significant group effect (*p* = 0.0018; ε^2^ = 0.156, indicating a moderate-to-large effect size). Dunn post hoc analysis with Holm correction revealed that both HP-4 and HP-1 groups exhibited significantly lower None values compared with untreated controls (*p* = 0.0011 and *p* = 0.0052, respectively) (Table 3).

Descriptive analyses supported these findings, with the untreated group demonstrating the highest proportion of non-melanized beads (34.5%), whereas HP-4 and HP-1 showed markedly lower values (6.8% and 8.0%, respectively) (Table 3).

Significant differences were also detected for melanized bead proportions (ANOVA *p* = 0.00014; η^2^ = 0.238, indicating a large effect size). Tukey post hoc analyses demonstrated that all HP treatment groups (HP-1, HP-2, HP-3, and HP-4) exhibited significantly higher melanization rates compared with untreated controls (all *p* < 0.015). In contrast, the DW group did not differ significantly from untreated controls. However, no statistically significant differences were observed among treatment groups themselves (Table 3).

Descriptive analyses demonstrated consistently elevated melanization responses across all injected groups, with melanized bead percentages ranging from 87.9% to 93.2%, compared with 65.4% in untreated larvae (Table 3).

## 4. Discussion

This study systematically evaluated the effects of *H. perforatum* extract on the interaction between innate immune responses and oxidative homeostasis in the *G. mellonella* model. The present findings demonstrated that the extract induced significant alterations in hemolymph total protein levels, total hemocyte count (THC), encapsulation responses, and melanization dynamics, whereas antioxidant enzyme activities and lipid peroxidation markers remained largely stable under the evaluated experimental conditions. Collectively, these findings suggest that *H. perforatum* may modulate both cellular and humoral immune functions without inducing detectable oxidative disruption during acute exposure.

One of the most notable findings of the study was the significant reduction in total hemolymph protein levels observed in the HP-2 group. In insect physiology, alterations in the hemolymph protein pool are frequently associated with redistribution of metabolic resources toward defense-related pathways [36]. Considering that the same dose was previously shown to enhance antimicrobial peptide expression in the same experimental model [16], the observed reduction in total protein levels may reflect an immunometabolic trade-off mechanism rather than generalized metabolic suppression. Under this framework, the relative abundance of immune effector proteins may increase despite a reduction in total circulating protein content. This interpretation is consistent with evolutionary and immunometabolic models proposing that immune activation requires substantial energetic investment and coordinated metabolic reorganization [37,38]. The dissociation between total protein reduction and the absence of overt oxidative damage further supports the possibility that the observed response represents adaptive physiological reallocation rather than cytotoxic stress.

The present study additionally demonstrated that *H. perforatum* exposure significantly altered hemocyte dynamics in a dose-dependent manner. THC analysis revealed the highest hemocyte levels in the HP-2 group, whereas higher concentrations (HP-3 and HP-4) were associated with values approaching control levels. These findings suggest a biphasic or hormetic-like immunomodulatory profile, in which intermediate–low concentrations promote immune activation while higher concentrations may limit cellular immune expansion. *G. mellonella* hemocytes play central roles in both humoral and cellular immune responses, including phagocytosis, encapsulation, melanization, and antimicrobial peptide regulation [17]. Therefore, the increase observed in the HP-2 group may indicate enhanced hemocyte mobilization, proliferation, or hematopoietic activity following immune stimulation. Similar hemocyte activation patterns have been described following exposure to pathogen-associated or phytochemical immune stimuli in insect models [19,21,39].

In contrast, the absence of further THC elevation at higher concentrations may indicate the emergence of regulatory or stress-associated mechanisms limiting hemocyte homeostasis. Several studies have demonstrated that phytochemical compounds, including hyperforin-related metabolites, may influence mitochondrial activity, apoptosis-associated pathways, and cellular survival signaling depending on concentration and exposure conditions [40,41]. Therefore, the relatively lower THC values observed in HP-3 and HP-4 groups may reflect alterations in hemocyte survival, mobilization efficiency, or hematopoietic regulation. However, because apoptotic markers and hemocyte subtype analyses were not directly evaluated in the current study, these mechanistic interpretations remain speculative and require further molecular validation.

Encapsulation analyses further supported the existence of concentration-dependent immune modulation. Encapsulation represents one of the principal cellular defense mechanisms in insects and reflects the ability of hemocytes to organize coordinated multilayer responses against foreign structures. In the present study, the HP-2 group consistently maintained stronger encapsulation responses at both 4 h and 24 h, whereas the HP-3 and particularly the HP-4 groups demonstrated increased weak encapsulation profiles. The coexistence of elevated THC values and enhanced strong encapsulation responses in the HP-2 group suggests coordinated activation of cellular immune defense mechanisms rather than isolated hemocyte proliferation alone. Previous studies have shown that natural and synthetic immunomodulators may influence hemocyte adhesion, spreading, aggregation, and capsule organization [42,43]. In this context, the HP-2 dose may have promoted more effective hemocyte coordination and foreign-body recognition processes.

Conversely, the predominance of weak encapsulation responses in the HP-4 group suggests that higher extract concentrations may impair the structural organization of encapsulation rather than completely suppressing immune activation. This distinction is biologically important because weak encapsulation may reflect incomplete hemocyte coordination, altered adhesion capacity, or dysregulated capsule maturation. The persistence of this pattern at 24 h indicates that these alterations are not limited to an early transient response but may represent sustained functional modulation of cellular immunity. Accordingly, the present findings suggest that increasing extract concentrations may shift the immune response from coordinated cellular defense toward a less organized immunophysiological profile.

Melanization analyses provided additional insight into the humoral immune effects of *H. perforatum*. Melanization is a major innate defense mechanism mediated through activation of the prophenoloxidase (proPO) cascade and contributes to pathogen immobilization, wound repair, and oxidative immune defense [8,12,44]. In the current study, no statistically significant differences were observed at the 4 h time point, suggesting that early melanization responses remained relatively similar across groups. This observation may indicate that the initial phase following injection is dominated primarily by foreign-body recognition and early immune activation rather than fully established melanization responses.

In contrast, substantial differences emerged at 24 h. All injected groups demonstrated significantly increased melanization compared with untreated controls, indicating that both extract exposure and injection-related injury may contribute to proPO system activation. Sterile injury itself is known to induce phenoloxidase activation and melanization in insects [18,26]. However, the immune profile observed in the HP-4 group was particularly notable when interpreted together with encapsulation findings. Although HP-4 exhibited reduced strong encapsulation responses, melanization remained markedly elevated. This apparent dissociation between cellular organization and humoral activation suggests that high extract concentrations may differentially regulate distinct immune compartments. One possible explanation is that impaired hemocyte coordination at high doses may be partially compensated by sustained or enhanced proPO-mediated humoral defense activity. Similar compensatory relationships between encapsulation efficiency and melanization intensity have previously been described in insect immune systems [7,26].

Among all treatment groups, HP-2 appeared to produce the most coordinated immune phenotype, characterized by elevated THC levels, stronger encapsulation responses, and increased melanization tendency. Together, these findings suggest simultaneous activation of both cellular and humoral immune components at intermediate–low extract concentrations. In contrast, HP-1 primarily enhanced melanization without substantial THC elevation, indicating that melanization dynamics may depend not only on hemocyte abundance but also on functional activation of the proPO system. Previous studies performed in the same *G. mellonella*–Hypericum model have similarly demonstrated dose-dependent modulation of phenoloxidase activity and antimicrobial peptide gene expression [16,17]. These observations collectively support the hypothesis that *H. perforatum* may be associated with concentration-dependent immunomodulatory patterns capable of generating distinct immune phenotypes depending on exposure concentration.

From the perspective of oxidative homeostasis, the stability of CAT, SOD, and MDA values across all groups suggests that acute extract exposure did not induce measurable oxidative injury under the tested conditions [45,46,47]. This observation is particularly important because activation of innate immunity is often accompanied by reactive oxygen species production and oxidative stress-related tissue damage. The preservation of antioxidant enzyme stability despite substantial immune modulation suggests that the observed physiological responses occurred within a controlled immunophysiological range rather than under overt oxidative toxicity. Similarly, unchanged MDA levels indicate that membrane lipid peroxidation did not occur to a detectable extent during the evaluated period. These findings are consistent with the hypothesis that some plant-derived immunomodulators may influence immune signaling without necessarily disrupting systemic redox balance.

Previous studies investigating different botanical extracts in insect models have produced variable oxidative stress profiles. For example, *Olea europaea* extracts were reported to suppress antioxidant enzyme activities while increasing MDA levels [48]. Similarly, *H. perforatum* essential oil exposure in *Tenebrio molitor* was associated with reductions in SOD, CAT, and GPx activities together with increased lipid peroxidation [49]. In contrast, mammalian studies frequently report antioxidant and hepatoprotective effects of flavonoid-rich *H. perforatum* extracts, including increased CAT activity and reduced oxidative stress markers [50]. These discrepancies likely arise from differences in phytochemical composition, extraction methods, exposure duration, administration routes, metabolic organization, detoxification systems, and species-specific immune physiology. Therefore, the absence of detectable oxidative disruption in the present study should be interpreted within the specific biological and experimental context of the acute *G. mellonella* model rather than generalized across systems.

The qualitative GC–MS analysis identified phenolic derivatives, fatty acid-related compounds, and heterocyclic metabolites potentially associated with immunological and redox-related biological activity. Although these analyses do not permit direct quantification or definitive compound identification, the detected phytochemical profile provides a plausible biochemical basis for the observed immune-associated physiological responses. Importantly, the current findings emphasize that the biological effects of phytochemicals cannot be predicted solely on the basis of their in vitro antioxidant capacity. Instead, immune regulation, metabolic adaptation, and systemic physiological context appear to be critical determinants of in vivo responses.

Finally, the present study highlights the value of *G. mellonella* as an alternative experimental model for investigating botanical immunomodulators under controlled conditions. Because developing organisms may exhibit distinct metabolic and oxidative regulatory profiles [51,52], experimental systems capable of simultaneously evaluating immune and oxidative responses may provide useful preliminary mechanistic insights. Given the increasing use of complementary herbal products in pediatric and general populations, understanding the immunophysiological effects of plant-derived compounds remains increasingly relevant [53,54,55,56]. Nevertheless, direct translational interpretation should be approached cautiously, and further validation in vertebrate systems together with integrative transcriptomic, proteomic, and metabolomic analyses will be required to clarify the molecular basis of the observed responses.

## 5. Limitations

Several limitations of the present study should be considered when interpreting the findings. First, although total hemocyte count and functional immune responses were evaluated, hemocyte subtypes were not characterized separately. Because different hemocyte populations may exhibit distinct immunological functions, additional subtype-specific analyses could provide more detailed insight into the cellular mechanisms underlying the observed responses.

Second, apoptotic activity, mitochondrial function, and direct cell viability parameters were not assessed. Therefore, the mechanisms potentially responsible for the altered hemocyte dynamics observed at higher extract concentrations remain speculative.

Third, the GC–MS analysis performed in this study represented a preliminary qualitative phytochemical screening rather than definitive quantitative compound characterization. Consequently, direct attribution of the observed biological responses to specific phytochemical constituents should be approached cautiously. In addition, transcriptomic, proteomic, and metabolomic analyses were not performed; therefore, the molecular pathways associated with the observed immunophysiological alterations could not be comprehensively evaluated.

Another important limitation is that only acute exposure conditions were investigated. Longer-term exposure studies may reveal additional adaptive, compensatory, or cumulative physiological effects that were not detectable within the 24 h experimental period. Finally, although *G. mellonella* provides important advantages as a 3R-compliant alternative experimental model with conserved innate immune mechanisms, direct extrapolation of the present findings to vertebrate or clinical systems remains limited. Accordingly, further validation studies using vertebrate models and integrative molecular approaches are required to clarify the translational relevance of these findings.

## 6. Conclusions

In conclusion, *H. perforatum* extract induced dose-dependent immune modulation in the *G. mellonella* model under acute exposure conditions. Intermediate–low concentrations, particularly HP-2, were associated with coordinated activation of cellular immune responses, including increased hemocyte abundance and enhanced encapsulation activity, whereas higher concentrations promoted weaker encapsulation profiles and altered immune organization.

Despite these immunophysiological alterations, antioxidant enzyme activities and lipid peroxidation markers remained stable, suggesting that the observed responses occurred without detectable oxidative disruption under the evaluated conditions. Collectively, these findings indicate that *H. perforatum* may differentially regulate cellular and humoral innate immune mechanisms in a concentration-dependent manner.

The present study further highlights the utility of *G. mellonella* as an alternative experimental platform for investigating botanical immunomodulators. However, additional transcriptomic, proteomic, metabolomic, and vertebrate-based studies are required to clarify the molecular mechanisms and translational relevance underlying these responses.

## Figures and Tables

**Table 1:** Tentative identification of bioactive compounds in *H. perforatum* extract using GC-MS analysis.

RT (min)	Tentatively Identified Compound	Molecular Formula	MW (g/mol)	Area (%)	Spectral Match (SI)	Putative Biological Relevance
18.20	9,12,15-Octadecatrienoic acid derivative (TMS ester)	C27H52O4Si2	496	6.60	429–532	Unsaturated fatty acid derivative; antioxidant/lipid-modulating
42.11	3-(1H-Indazol-6-ylamino)-6,7-dimethoxy-3H-isobenzofuran-1-one	C17H15N3O4	325	10.12	503–596	Phenolic/heterocyclic derivative
48.26	Benzoic acid, 3,4,5-trihydroxy-, propyl ester	C10H12O5	212	6.90	514–621	Phenolic antioxidant (gallate derivative)
53.17	Benzoic acid, 3,4,5-trihydroxy-, propyl ester	C10H12O5	212	6.41	498–618	Phenolic antioxidant
53.94	Benzoic acid, 3,4,5-trihydroxy-, propyl ester	C10H12O5	212	5.98	517–661	Phenolic antioxidant

**Table 2:** Effects of *H. perforatum* extract on encapsulation responses in *G. mellonella* larvae at 4 h and 24 h following Sephadex bead injection.

4 h (%) Mean ± SE *			
Group	None	Weak	Strong
Untreated	4.92 ± 1.34 ^a^	70.8 ± 3.48 ^ab^	24.2 ± 3.47 ^ab^
DW	5.17 ± 1.16 ^a^	71.6 ± 2.42 ^b^	23.3 ± 2.26 ^ab^
HP-1	3.86 ± 1.07 ^a^	73.9 ± 3.68 ^ab^	22.2 ± 3.36 ^ab^
HP-2	1.82 ± 0.63 ^a^	64.1 ± 5.47 ^b^	34.1 ± 5.53 ^b^
HP-3	3.87 ± 1.17 ^a^	85.0 ± 2.28 ^a^	11.2 ± 2.65 ^a^
HP-4	6.87 ± 1.59 ^a^	78.6 ± 2.67 ^ab^	14.5 ± 2.94 ^a^
24 h (%) Mean ± SE *			
Untreated	4.08 ± 1.07 ^a^	43.3 ± 1.51 ^a^	52.6 ± 1.38 ^b^
DW	3.84 ± 1.21 ^a^	41.4 ± 1.39 ^a^	54.8 ± 1.79 ^b^
HP-1	0.96 ± 0.53 ^a^	39.2 ± 1.00 ^a^	59.8 ± 1.15 ^c^
HP-2	3.20 ± 1.65 ^a^	40.4 ± 2.46 ^a^	56.5 ± 1.40 ^b^
HP-3	2.23 ± 0.70 ^a^	51.0 ± 2.30 ^b^	46.8 ± 2.87 ^b^
HP-4	1.34 ± 0.62 ^a^	66.8 ± 3.53 ^b^	31.8 ± 3.56 ^a^

* Each value represents the mean ± standard error (SE) of 16 replicates. Different letters within the same column indicate statistically significant differences between groups (One-way ANOVA, Tukey’s HSD, *p* < 0.05).

**Table 3:** Effects of *H. perforatum* extract on melanization responses in *G. mellonella* larvae at 4 h and 24 h following Sephadex bead injection.

4 h (%) Mean ± SE *		
Group	Non-melanized	Melanized
Untreated	28.93 ± 4.31 ^a^	71.07 ± 4.31 ^a^
DW	27.90 ± 2.65 ^a^	72.10 ± 2.65 ^a^
HP-1	29.21 ± 4.40 ^a^	70.79 ± 4.40 ^a^
HP-2	18.65 ± 2.73 ^a^	81.35 ± 2.73 ^a^
HP-3	33.45 ± 5.32 ^a^	66.55 ± 5.32 ^a^
HP-4	31.54 ± 5.02 ^a^	68.46 ± 5.02 ^a^
24 h (%) Mean ± SE *		
Untreated	34.55 ± 5.59 ^a^	65.45 ± 5.59 ^a^
DW	30.54 ± 1.78 ^a^	69.46 ± 1.78 ^a^
HP-1	8.06 ± 1.91 ^b^	91.94 ± 1.91 ^b^
HP-2	12.10 ± 2.73 ^a^	87.90 ± 2.73 ^b^
HP-3	11.41 ± 2.04 ^a^	88.59 ± 2.04 ^b^
HP-4	6.80 ± 1.70 ^b^	93.20 ± 1.70 ^b^

* Each value represents the mean ± standard error (SE) of 16 replicates. Different letters within the same column indicate statistically significant differences between groups (*p* < 0.05). For 24 h melanized values, one-way ANOVA with Tukey’s HSD was used; for 24 h non-melanized values, Kruskal–Wallis with Dunn’s post hoc (Holm correction) was used. No significant differences were detected at 4 h for either variable.

## Data Availability

The data presented in this study are available within the article and Supplementary Materials, as well as in a public repository (Zenodo) at: https://doi.org/10.5281/zenodo.20439185.

## Supplementary Materials

The following supporting information can be downloaded at: https://www.mdpi.com/article/10.3390/biomedicines14061297/s1, Supplementary Material S1: ESOGU-ARUM; Supplementary Material S2: Table S1: Statistical analysis of total protein (TP) levels; Table S2: Statistical analysis of catalase (CAT) activity; Table S3: Statistical analysis of superoxide dismutase (SOD) activity; Table S4: Statistical analysis of malondialdehyde (MDA) levels; Table S5: Summary of parametric and non-parametric test results for each 4 h encapsulation variable; Table S6: Summary of non-parametric test results for each 24 h encapsulation variable; Table S7: Statistical Analysis of Melanization Responses at 4 H Descriptive statistics (mean ± SD) after asin(sqrt(percentage)); Table S8: Statistical Analysis of Melanization Responses at 24 H Descriptive statistics (mean ± SD) after asin(sqrt(percentage)); Table S9: Statistical Analysis THC results.

## Abbreviations

The following abbreviations are used in this manuscript:

GC–MS	Gas Chromatography–Mass Spectrometry
3R-compliant	Replacement, Reduction, and Refinement in animal research
ANOVA	Analysis of Variance
THC	Total Hemocyte Count
