# Supplementary material for: Antioxidant, Redox, and Immunomodulatory Effects of Hypericum perforatum in the Galleria mellonella: A 3R-Oriented Invertebrate Model
Source: Biomedicines. 2026 Jun 7;14(6):1297. doi: 10.3390/biomedicines14061297 (PMC13296390; doi:10.3390/biomedicines14061297)
Supplement: Supplementary file 1 [file biomedicines-14-01297-s001.zip › Supplementary Material S1.pdf]

ESOGU-ARUM

Data File:	111858_7	Original Data Path:	C:\Xcalibur\data\OKN
Current Data Path:	C:\Xcalibur\data\OKN	Sample Type:	Unknown
Sample ID:	1	Acquisition Date:	04/11/23 08:12:02 AM
Run Time(min):	57.02	Vial:	18

Instrument Method:	C:\Xcalibur\methods\xMED.meth
Original Processing Method:	C:\Xcalibur\methods\arum_xx_qual
Current Processing Method:	N/A

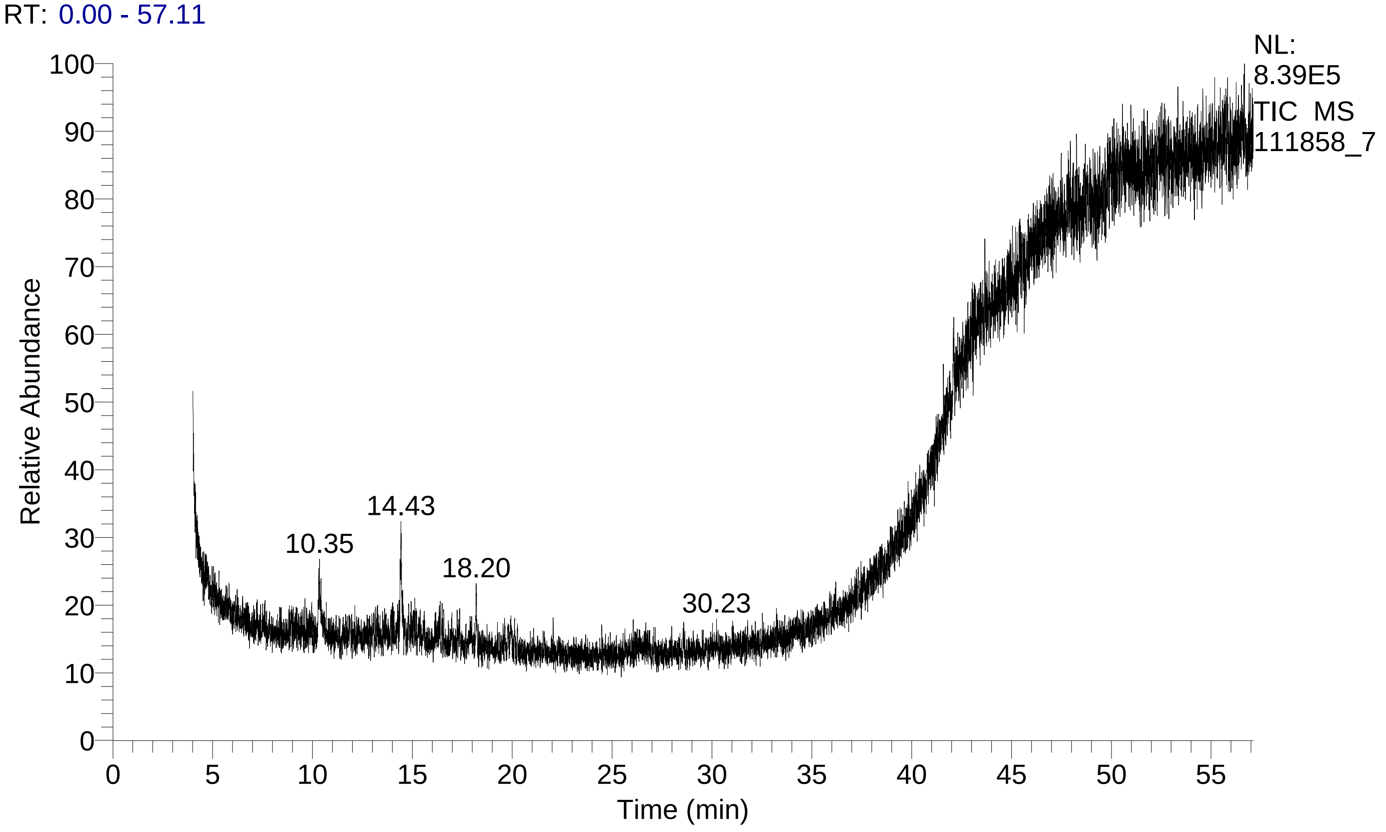

There is no signature data to report.

ESOGU-ARUM

Hit Spectrum	Compound Structure

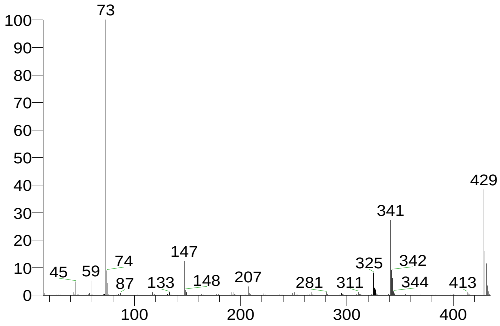

Cyclohexasiloxane, dodecamethyl-
Formula C12H36O6Si6, MW 444, CAS# 540-97-6, Entry# 37894
Dodecamethylcyclohexasiloxane

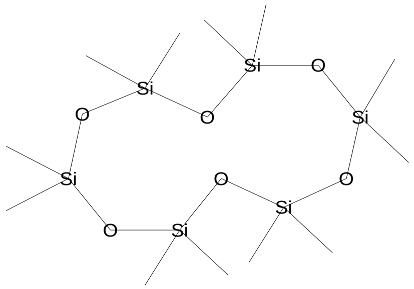

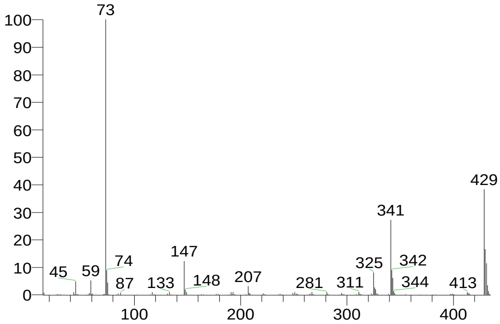

Cyclohexasiloxane, dodecamethyl- (CAS)
Formula C12H36O6Si6, MW 444, CAS# 540-97-6, Entry# 584464
Dodecamethylcyclohexasiloxane

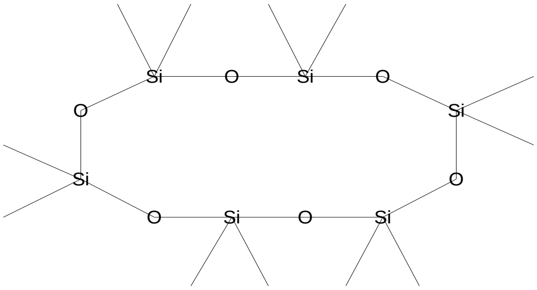

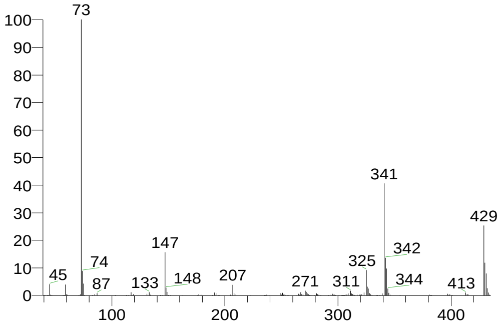

CYCLOHEXASILOXANE, DODECAMETHYL-
Formula C12H36O6Si6, MW 444, CAS# 540-97-6, Entry# 584463
2,2,4,4,6,6,8,8,10,10,12,12-DODECAMETHYLCYCLOHEXASILOXANE #

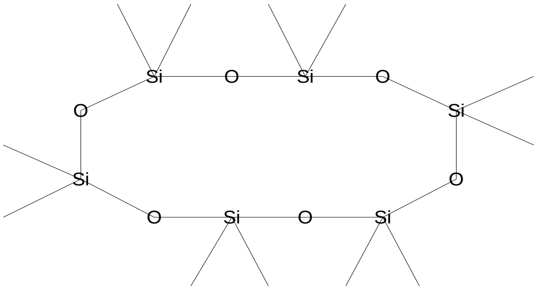

RT	Probability	Compound Name	SI	RSI	Cas #	Area %	Area
10.35	19.54	Cyclohexasiloxane, dodecamethyl-	446	675	540-97-6	9.80	367019.64
10.35	19.54	Cyclohexasiloxane, dodecamethyl- (CAS)	446	673	540-97-6	9.80	367019.64
10.35	19.54	CYCLOHEXASILOXANE, DODECAMETHYL-	443	683	540-97-6	9.80	367019.64

There is no signature data to report.

ESOGU-ARUM

Hit Spectrum	Compound Structure

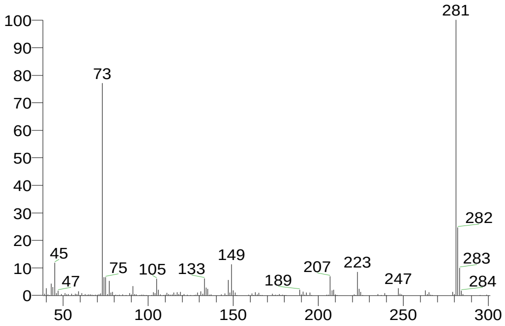

Benzoic acid, 3-methyl-2-trimethylsilyloxy-, trimethylsilyl ester
Formula C14H24O3Si2, MW 296, CAS# NA, Entry# 173822

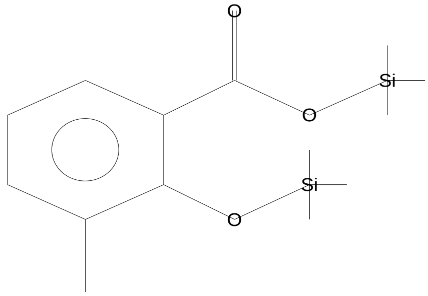

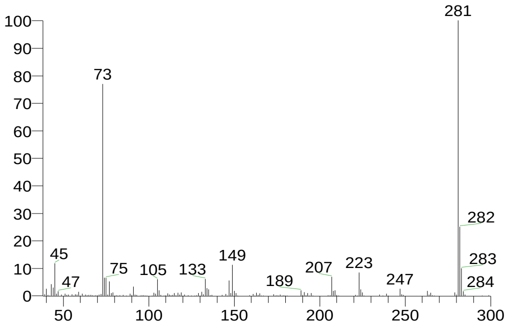

TRIMETHYLSILYL ESTER OF 3-METHYL-2-TRIMETHYLSILYLOXY-BENZOIC ACID
Formula C14H24O3Si2, MW 296, CAS# NA, Entry# 364618

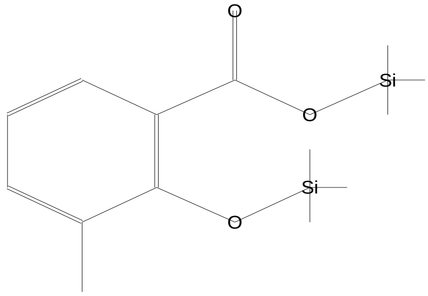

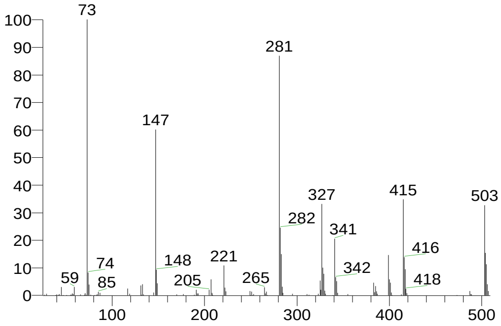

Cycloheptasiloxane, tetradecamethyl-
Formula C14H42O7Si7, MW 518, CAS# 107-50-6, Entry# 37487
2,2,4,4,6,6,8,8,10,10,12,12,14,14-Tetradecamethylcycloheptasiloxane #

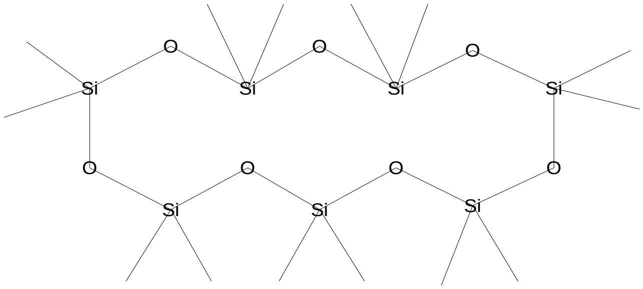

RT	Probability	Compound Name	SI	RSI	Cas #	Area %	Area
14.43	6.91	Benzoic acid, 3-methyl-2-trimethylsilyloxy-, trimethylsilyl ester	451	577	NA	18.14	679416.89
14.43	6.91	TRIMETHYLSILYL ESTER OF 3-METHYL-2-TRIMETHYLSILYLOXY-BENZOIC ACID	451	576	NA	18.14	679416.89
14.43	5.15	Cycloheptasiloxane, tetradecamethyl-	443	596	107-50-6	18.14	679416.89

There is no signature data to report.

ESOGU-ARUM

Hit Spectrum | Compound Structure

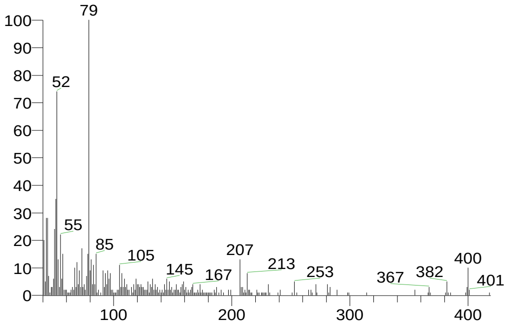

Pregnane-3,11,20,21-tetrol, cyclic 20,21-(butyl boronate), (3à,5á,11á,20R)-
Formula C25H43BO4, MW 418, CAS# 55556-74-6, Entry# 41592
17-(2-Butyl-1,3,2-dioxaborolan-4-yl)androstane-3,11-diol #

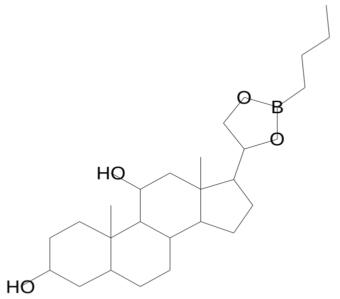

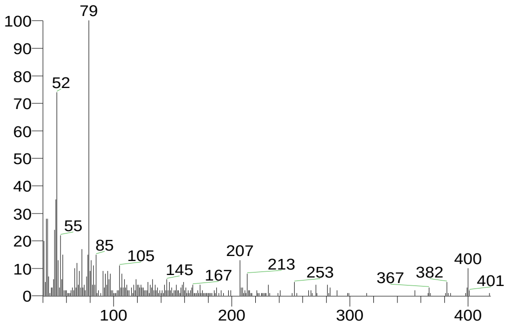

Pregnane-3,11,20,21-tetrol, cyclic 20,21-(butyl boronate), (3à,5á,11á,20R)- (CAS)
Formula C25H43BO4, MW 418, CAS# 55556-74-6, Entry# 562492
5á-PREGNAN-3à,11á,20à,21-TETROL BUTYL BORONATE

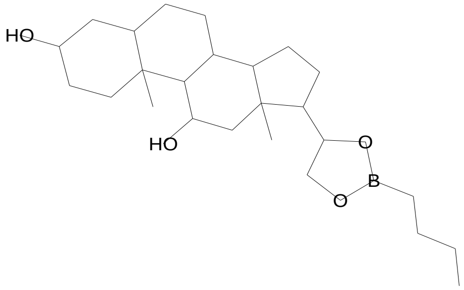

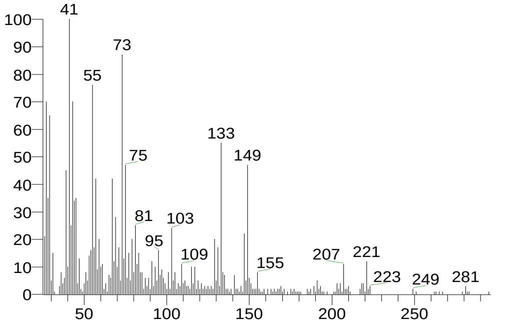

9,12,15-Octadecatrienoic acid, 2-[(trimethylsilyl)oxy]-1-[[(trimethylsilyl)oxy]methyl]ethyl ester, (Z,Z,Z)-
Formula C27H52O4Si2, MW 496, CAS# 55521-23-8, Entry# 3312
2-[(Trimethylsilyl)oxy]-1-([(trimethylsilyl)oxy]methyl)ethyl (9E,12E,15E)-9,12,15-octadecatrienoate #

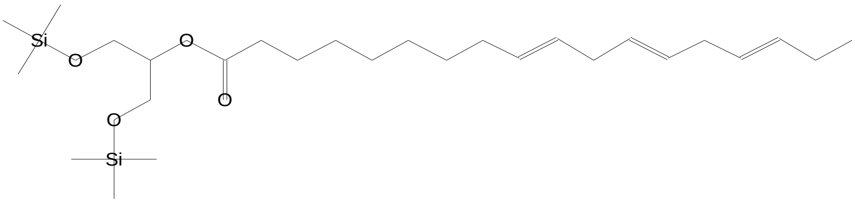

RT	Probability	Compound Name	SI	RSI	Cas #	Area %	Area
18.20	9.02	Pregnane-3,11,20,21-tetrol, cyclic 20,21-(butyl boronate), (3à,5á,11á,20R)-	435	497	55556-74-6	6.60	247113.44
18.20	9.02	Pregnane-3,11,20,21-tetrol, cyclic 20,21-(butyl boronate), (3à,5á,11á,20R)- (CAS)	435	497	55556-74-6	6.60	247113.44
18.20	7.09	9,12,15-Octadecatrienoic acid, 2-[(trimethylsilyl)oxy]-1-[[(trimethylsilyl)oxy]methyl]ethyl ester, (Z,Z,Z)-	429	532	55521-23-8	6.60	247113.44

There is no signature data to report.

ESOGU-ARUM

Hit Spectrum	Compound Structure

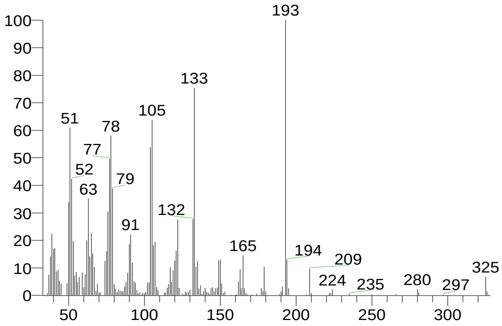

3-(1H-INDAZOL-6-YLAMINO)-6,7-DIMETHOXY-3H-ISOBENZOFURAN-1-ONE
Formula C17H15N3O4, MW 325, CAS# NA, Entry# 426046
3-(1H-INDAZOL-6-YLAMINO)-6,7-DIMETHOXY-2-BENZOFURAN-1(3H)-ONE

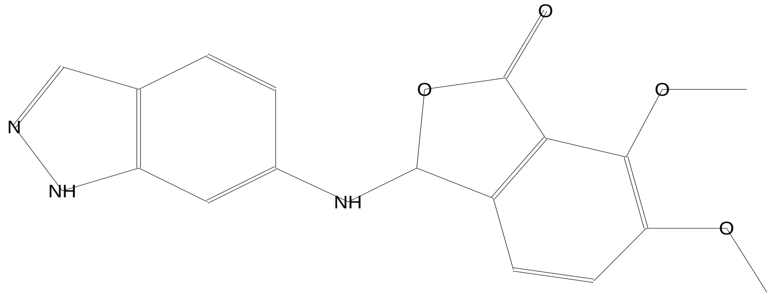

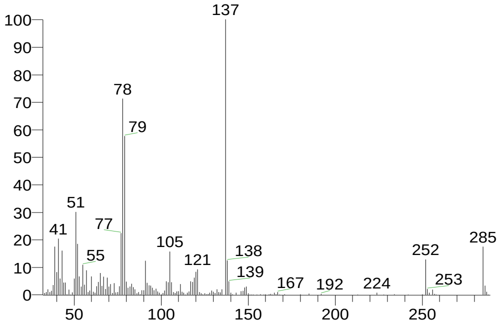

N-(2,5-Dimethyl-phenyl)-2-(pyridin-2-ylamino)-2-thioxo-acetamide
Formula C15H15N3OS, MW 285, CAS# NA, Entry# 340953
N-(2,5-Dimethylphenyl)-2-(2-pyridinylamino)-2-thioxoacetamide

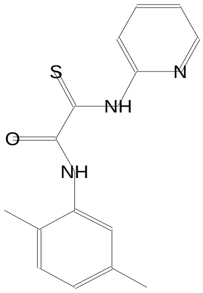

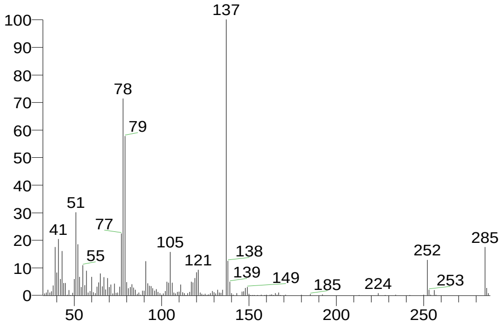

N-(2,5-Dimethyl-phenyl)-2-(pyridin-2-ylamino)-2-thioxo-acetamide
Formula C15H15N3OS, MW 285, CAS# NA, Entry# 99711

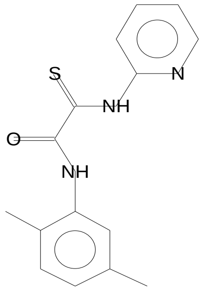

RT	Probability	Compound Name	SI	RSI	Cas #	Area %	Area
42.11	9.29	3-(1H-INDAZOL-6-YLAMINO)-6,7-DIMETHOXY-3H-ISOBENZOFURAN-1-ONE	503	596	NA	10.12	379308.16
42.11	7.85	N-(2,5-Dimethyl-phenyl)-2-(pyridin-2-ylamino)-2-thioxo-acetamide	499	612	NA	10.12	379308.16
42.11	6.93	N-(2,5-Dimethyl-phenyl)-2-(pyridin-2-ylamino)-2-thioxo-acetamide	496	609	NA	10.12	379308.16

There is no signature data to report.

ESOGU-ARUM

Hit Spectrum | Compound Structure

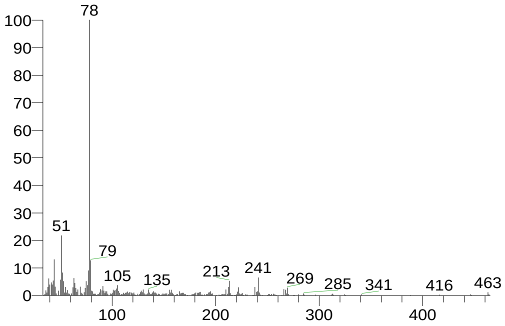

Formula C22H17N5O7, MW 463, CAS# NA, Entry# 597950

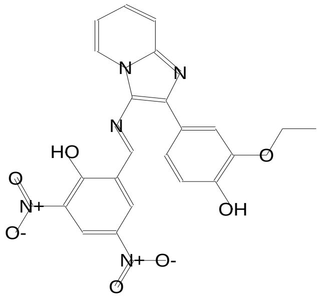

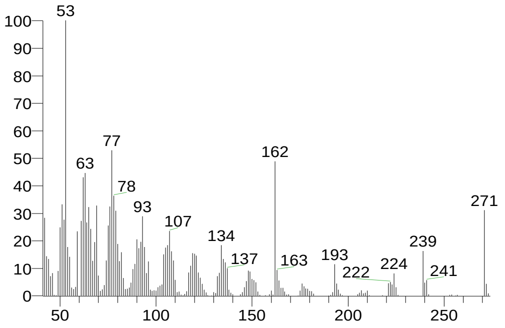

3,6-Dimethoxy-2,5-dinitrobenzaldehyde oxime
Formula C9H9N3O7, MW 271, CAS# NA, Entry# 16748

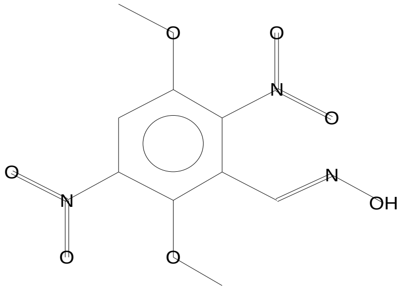

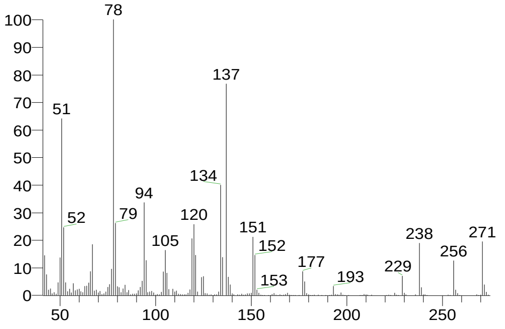

methyl 2-pyridyl ketone 4-[2-pyridyl]-3-thiosemicarbazone
Formula C13H13N5S, MW 271, CAS# 70619-05-5, Entry# 41366
(1E)-1-(3-Pyridinyl)ethanone N-(2-pyridinyl)thiosemicarbazone #

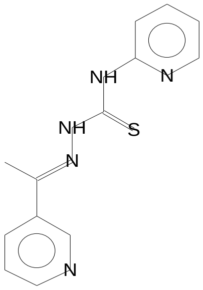

RT	Probability	Compound Name	SI	RSI	Cas #	Area %	Area
43.67	12.26	2-({[2-(3-ETHOXY-4-HYDROXYPHENYL)IMIDAZO[1,2-A]PYRIDIN-3-YL]IMINO}METHYL)-4,6-DINITROPHENOL	505	604	NA	6.25	234141.06
43.67	8.65	3,6-Dimethoxy-2,5-dinitrobenzaldehyde oxime	495	624	NA	6.25	234141.06
43.67	7.31	methyl 2-pyridyl ketone 4-[2-pyridyl]-3-thiosemicarbazone	491	596	70619-05-5	6.25	234141.06

There is no signature data to report.

ESOGU-ARUM

Hit Spectrum	Compound Structure

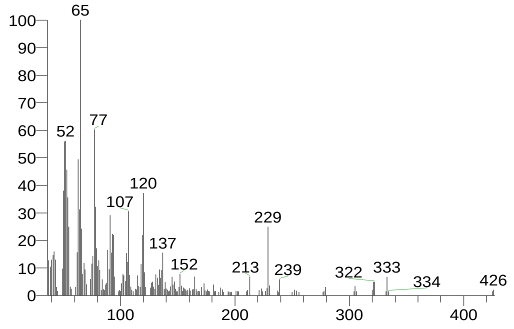

7,12-Dihydro-6,7-bis(4-hydroxyphenyl)-6H-[1,2,4]triazolo[1',5':1,2]pyrimido[5,4-c]chromen-2-ol
Formula C24H18N4O4, MW 426, CAS# NA, Entry# 27977

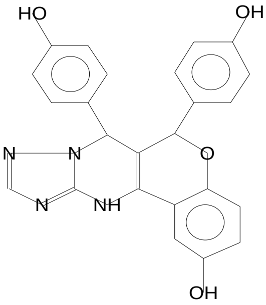

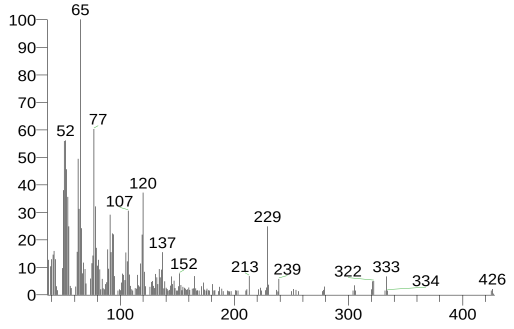

7,12-Dihydro-6,7-bis(4-hydroxyphenyl)-6H-[1,2,4]triazolo[1',5':1,2]pyrimido[5,4-c]chromen-2-ol
Formula C24H18N4O4, MW 426, CAS# NA, Entry# 569540
7,12-DIHYDRO-6,7-BIS(4-HYDROXYPHENYL)-6H-[1,2,4]TRIAZOLO[1',5':1,

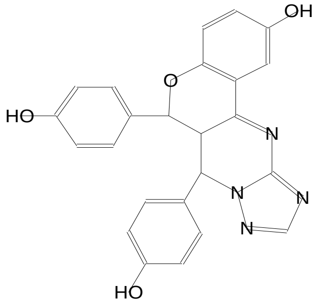

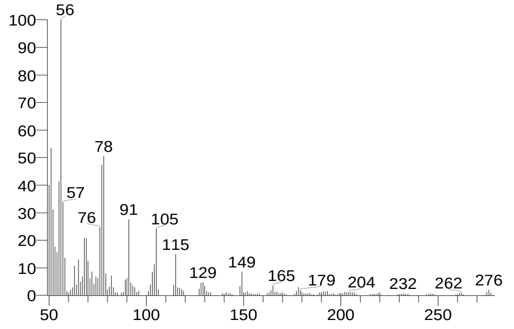

DIMETHYLDIPHENYLTETHYLIDYLPYRROLIDINE
Formula C20H23N, MW 277, CAS# NA, Entry# 323094

RT	Probability	Compound Name	SI	RSI	Cas #	Area %	Area
43.88	8.20	7,12-Dihydro-6,7-bis(4-hydroxyphenyl)-6H-[1,2,4]triazolo[1',5':1,2]pyrimido[5,4-c]chromen-2-ol	505	614	NA	4.39	164328.81
43.88	7.88	7,12-Dihydro-6,7-bis(4-hydroxyphenyl)-6H-[1,2,4]triazolo[1',5':1,2]pyrimido[5,4-c]chromen-2-ol	504	614	NA	4.39	164328.81
43.88	5.25	DIMETHYLDIPHENYLTETHYLIDYLPYRROLIDINE	492	642	NA	4.39	164328.81

There is no signature data to report.

ESOGU-ARUM

Hit Spectrum	Compound Structure

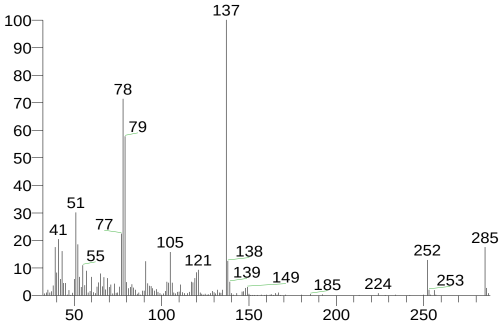

N-(2,5-Dimethyl-phenyl)-2-(pyridin-2-ylamino)-2-thioxo-acetamide
Formula C15H15N3OS, MW 285, CAS# NA, Entry# 99711

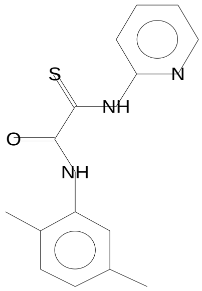

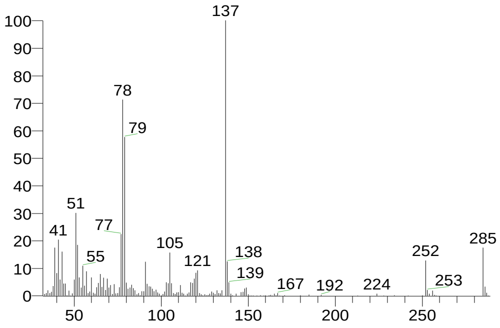

N-(2,5-Dimethyl-phenyl)-2-(pyridin-2-ylamino)-2-thioxo-acetamide
Formula C15H15N3OS, MW 285, CAS# NA, Entry# 340953
N-(2,5-Dimethylphenyl)-2-(2-pyridinylamino)-2-thioxoacetamide

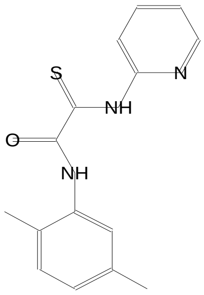

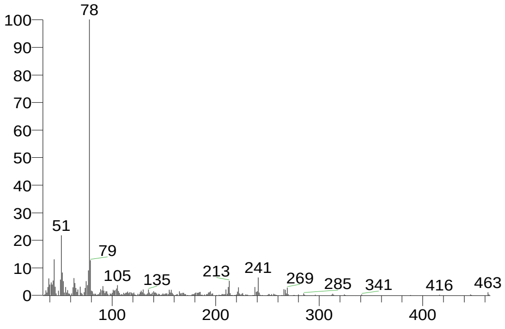

Formula C22H17N5O7, MW 463, CAS# NA, Entry# 597950

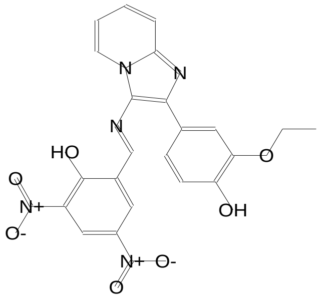

RT	Probability	Compound Name	SI	RSI	Cas #	Area %	Area
47.49	9.40	N-(2,5-Dimethyl-phenyl)-2-(pyridin-2-yl amino)-2-thioxo-acetamide	512	675	NA	4.26	159606.42
47.49	9.04	N-(2,5-Dimethyl-phenyl)-2-(pyridin-2-yl amino)-2-thioxo-acetamide	511	674	NA	4.26	159606.42
47.49	6.38	2-({[2-(3-ETHOXY-4-HYDROXYPHE NYL)IMIDAZO[1,2-A]PYRIDIN-3-YL]IMINO}METHYL)-4,6-DINITROPHE NOL	501	595	NA	4.26	159606.42

There is no signature data to report.

ESOGU-ARUM

Hit Spectrum	Compound Structure

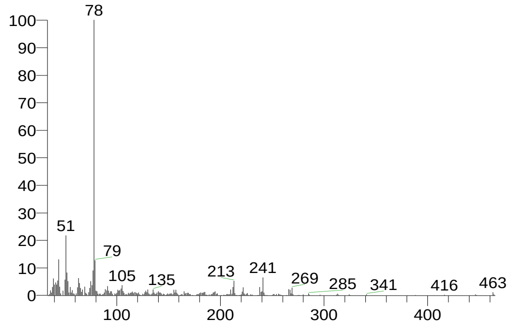

Formula C22H17N5O7, MW 463, CAS# NA, Entry# 597950

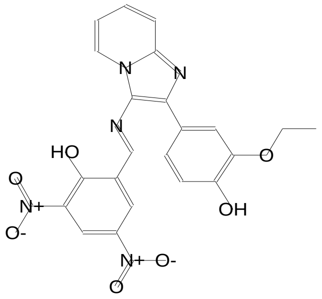

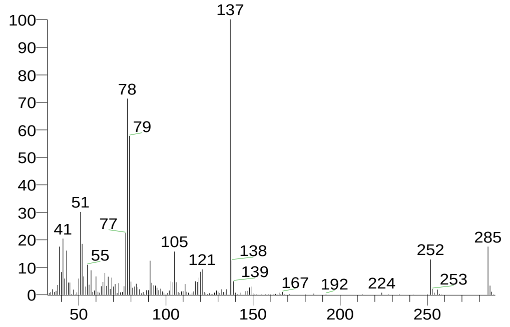

N-(2,5-Dimethyl-phenyl)-2-(pyridin-2-ylamino)-2-thioxo-acetamide
Formula C15H15N3OS, MW 285, CAS# NA, Entry# 340953
N-(2,5-Dimethylphenyl)-2-(2-pyridinylamino)-2-thioxoacetamide

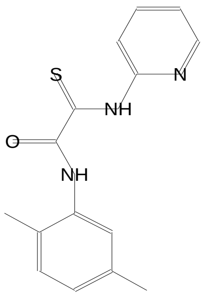

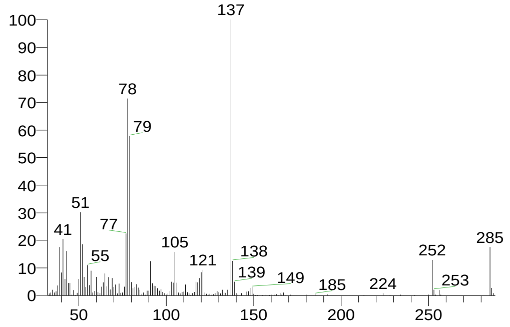

N-(2,5-Dimethyl-phenyl)-2-(pyridin-2-ylamino)-2-thioxo-acetamide
Formula C15H15N3OS, MW 285, CAS# NA, Entry# 99711

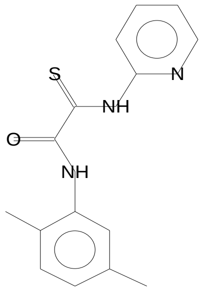

RT	Probability	Compound Name	SI	RSI	Cas #	Area %	Area
47.95	18.52	2-({[2-(3-ETHOXY-4-HYDROXYPHENYL)IMIDAZO[1,2-A]PYRIDIN-3-YL]IMINO}METHYL)-4,6-DINITROPHENOL	511	613	NA	3.62	135532.80
47.95	11.22	N-(2,5-Dimethyl-phenyl)-2-(pyridin-2-ylamino)-2-thioxo-acetamide	496	675	NA	3.62	135532.80
47.95	10.35	N-(2,5-Dimethyl-phenyl)-2-(pyridin-2-ylamino)-2-thioxo-acetamide	494	674	NA	3.62	135532.80

There is no signature data to report.

ESOGU-ARUM

Hit Spectrum	Compound Structure

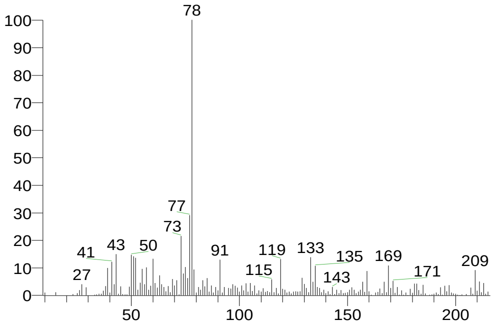

Benzoic acid, 3,4,5-trihydroxy-, propyl ester (CAS)
Formula C10H12O5, MW 212, CAS# 121-79-9, Entry# 168871
Nipa 49

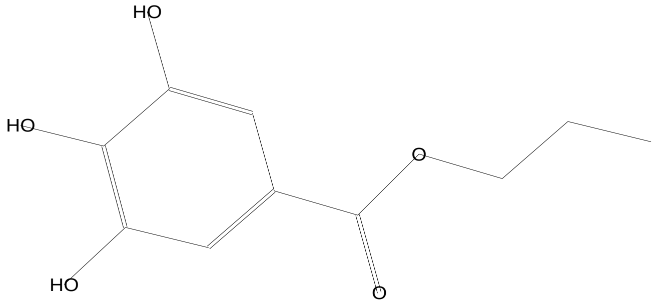

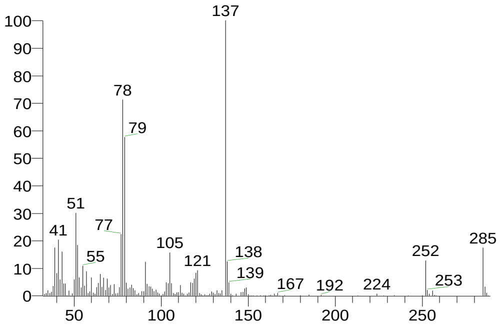

N-(2,5-Dimethyl-phenyl)-2-(pyridin-2-ylamino)-2-thioxo-acetamide
Formula C15H15N3OS, MW 285, CAS# NA, Entry# 340953
N-(2,5-Dimethylphenyl)-2-(2-pyridinylamino)-2-thioxoacetamide

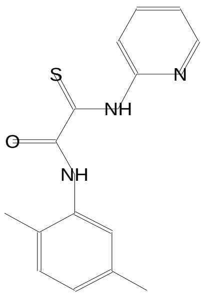

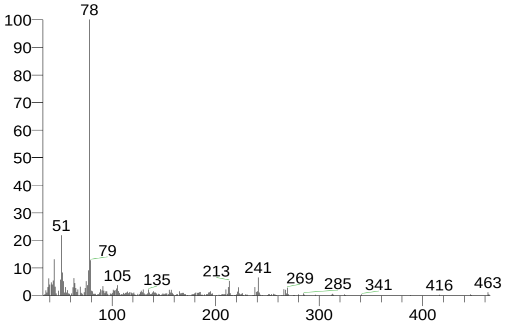

Formula C22H17N5O7, MW 463, CAS# NA, Entry# 597950

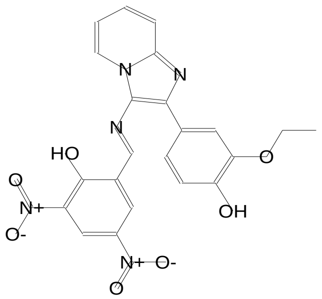

RT	Probability	Compound Name	SI	RSI	Cas #	Area %	Area
48.26	7.80	Benzoic acid, 3,4,5-trihydroxy-, propyl ester (CAS)	514	621	121-79-9	6.90	258352.68
48.26	5.50	N-(2,5-Dimethyl-phenyl)-2-(pyridin-2-yl amino)-2-thioxo-acetamide	504	651	NA	6.90	258352.68
48.26	5.08	2-({[2-(3-ETHOXY-4-HYDROXYPHE NYL)IMIDAZO[1,2-A]PYRIDIN-3-YL]IMINO}METHYL)-4,6-DINITROPHE NOL	502	581	NA	6.90	258352.68

There is no signature data to report.

ESOGU-ARUM

Hit Spectrum	Compound Structure

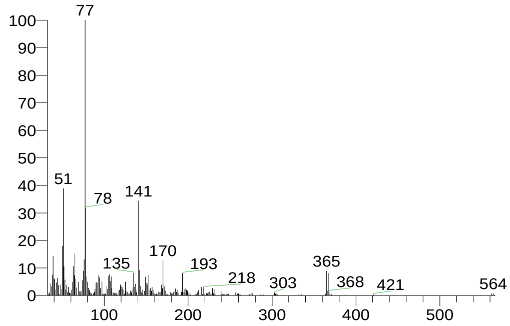

Formula C22H19BrN4O7S, MW 562, CAS# NA, Entry# 636813

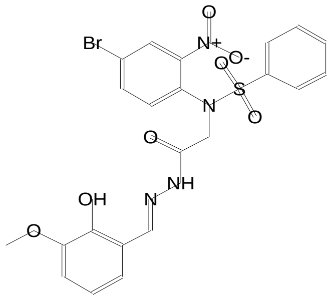

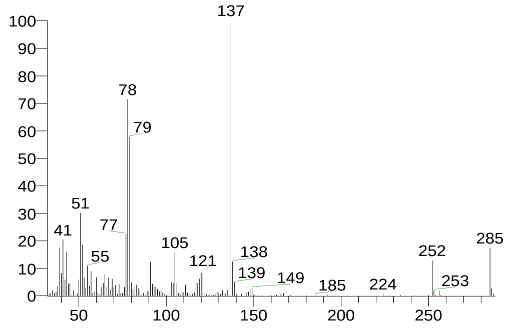

N-(2,5-Dimethyl-phenyl)-2-(pyridin-2-ylamino)-2-thioxo-acetamide
Formula C15H15N3OS, MW 285, CAS# NA, Entry# 99711

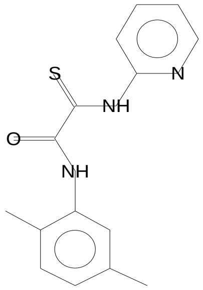

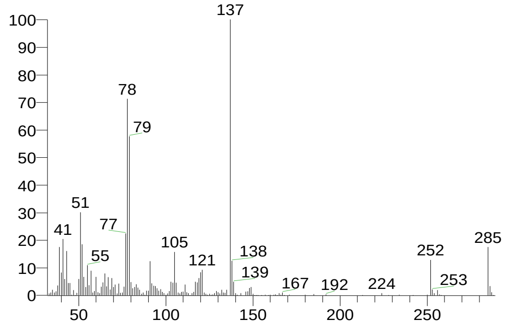

N-(2,5-Dimethyl-phenyl)-2-(pyridin-2-ylamino)-2-thioxo-acetamide
Formula C15H15N3OS, MW 285, CAS# NA, Entry# 340953
N-(2,5-Dimethylphenyl)-2-(2-pyridinylamino)-2-thioxoacetamide

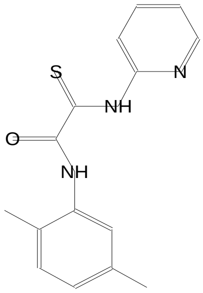

RT	Probability	Compound Name	SI	RSI	Cas #	Area %	Area
48.81	4.69	N-(4-BROMO-2-NITRO-PHENYL)-N-(2-HYDROXY-3-METHOXY-BENZYLIDENE-HYDRAZINOCARBONYLMETHYL)-BENZENESULFONAMIDE	460	567	NA	3.54	132514.84
48.81	3.96	N-(2,5-Dimethyl-phenyl)-2-(pyridin-2-ylamino)-2-thioxo-acetamide	456	630	NA	3.54	132514.84
48.81	3.96	N-(2,5-Dimethyl-phenyl)-2-(pyridin-2-ylamino)-2-thioxo-acetamide	456	628	NA	3.54	132514.84

There is no signature data to report.

ESOGU-ARUM

Hit Spectrum | Compound Structure

Formula C22H17N5O7, MW 463, CAS# NA, Entry# 597950

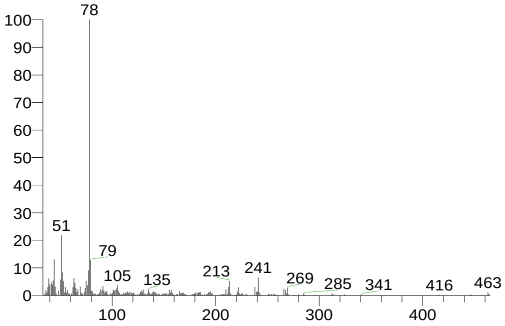

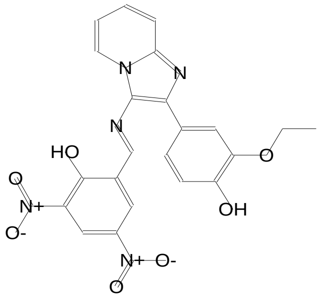

N-(2,5-Dimethyl-phenyl)-2-(pyridin-2-ylamino)-2-thioxo-acetamide
Formula C15H15N3OS, MW 285, CAS# NA, Entry# 99711

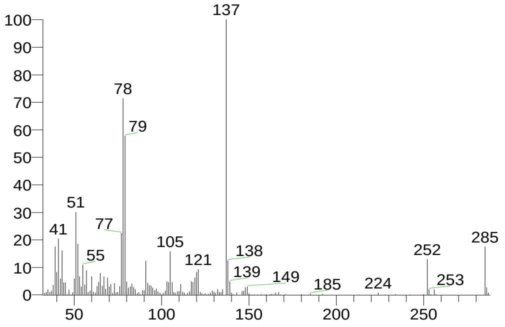

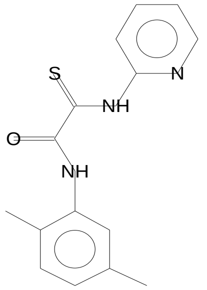

N-(2,5-Dimethyl-phenyl)-2-(pyridin-2-ylamino)-2-thioxo-acetamide
Formula C15H15N3OS, MW 285, CAS# NA, Entry# 340953
N-(2,5-Dimethylphenyl)-2-(2-pyridinylamino)-2-thioxoacetamide

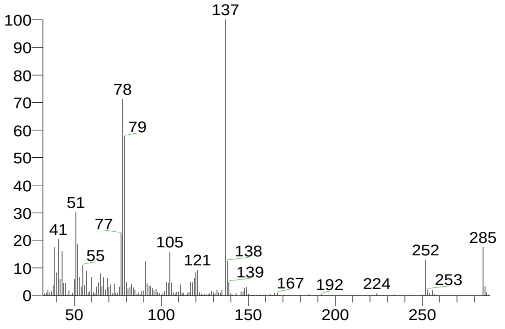

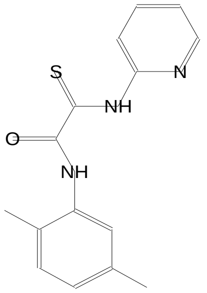

RT	Probability	Compound Name	SI	RSI	Cas #	Area %	Area
49.83	7.97	2-({[2-(3-ETHOXY-4-HYDROXYPHENYL)IMIDAZO[1,2-A]PYRIDIN-3-YL]IMINO}METHYL)-4,6-DINITROPHENOL	465	576	NA	6.47	242331.76
49.83	7.35	N-(2,5-Dimethyl-phenyl)-2-(pyridin-2-ylamino)-2-thioxo-acetamide	463	643	NA	6.47	242331.76
49.83	7.35	N-(2,5-Dimethyl-phenyl)-2-(pyridin-2-ylamino)-2-thioxo-acetamide	463	641	NA	6.47	242331.76

ESOGU-ARUM

Hit Spectrum	Compound Structure

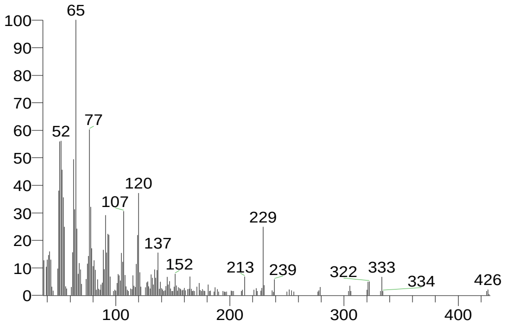

7,12-Dihydro-6,7-bis(4-hydroxyphenyl)-6H-[1,2,4]triazolo[1',5':1,2]pyrimido[5,4-c]chromen-2-ol
Formula C24H18N4O4, MW 426, CAS# NA, Entry# 569540
7,12-DIHYDRO-6,7-BIS(4-HYDROXYPHENYL)-6H-[1,2,4]TRIAZOLO[1',5':1,

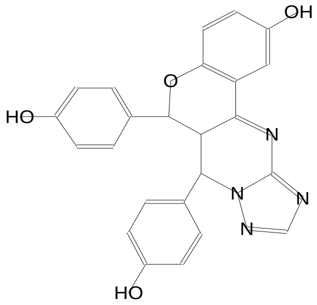

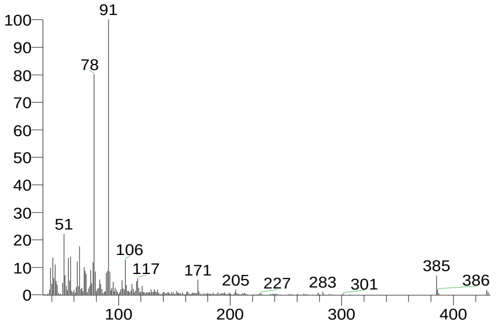

Formula C22H17N5O3S, MW 431, CAS# NA, Entry# 574442

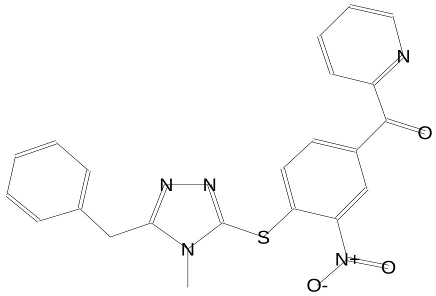

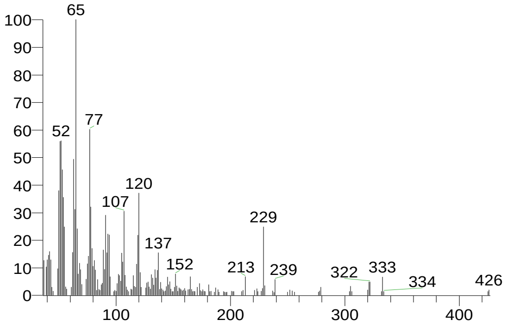

7,12-Dihydro-6,7-bis(4-hydroxyphenyl)-6H-[1,2,4]triazolo[1',5':1,2]pyrimido[5,4-c]chromen-2-ol
Formula C24H18N4O4, MW 426, CAS# NA, Entry# 27977

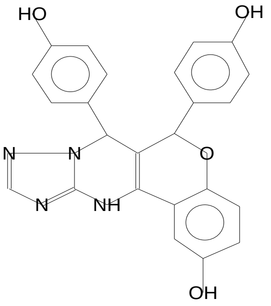

RT	Probability	Compound Name	SI	RSI	Cas #	Area %	Area
52.35	9.29	7,12-Dihydro-6,7-bis(4-hydroxyphenyl)-6H-[1,2,4]triazolo[1',5':1,2]pyrimido[5,4-c]chromen-2-ol	491	653	NA	4.47	167401.60
52.35	7.49	METHANONE, [4-[[4-METHYL-5-(PHENYLMETHYL)-4H-1,2,4-TRIAZOL-3-YL]THIO]-3-NITROPHENYL]2-PYRIDINYL-	486	580	NA	4.47	167401.60
52.35	7.19	7,12-Dihydro-6,7-bis(4-hydroxyphenyl)-6H-[1,2,4]triazolo[1',5':1,2]pyrimido[5,4-c]chromen-2-ol	485	648	NA	4.47	167401.60

There is no signature data to report.

ESOGU-ARUM

Hit Spectrum	Compound Structure

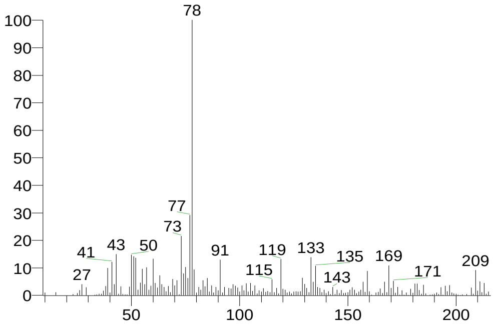

Benzoic acid, 3,4,5-trihydroxy-, propyl ester (CAS)
Formula C10H12O5, MW 212, CAS# 121-79-9, Entry# 168871
Nipa 49

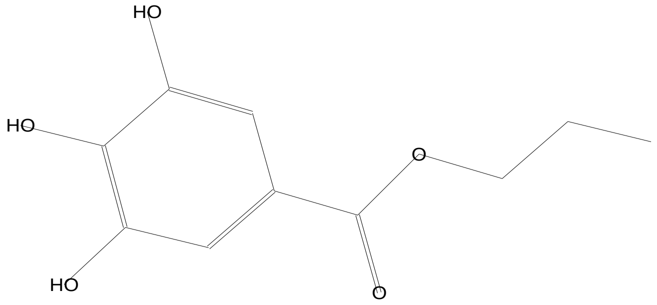

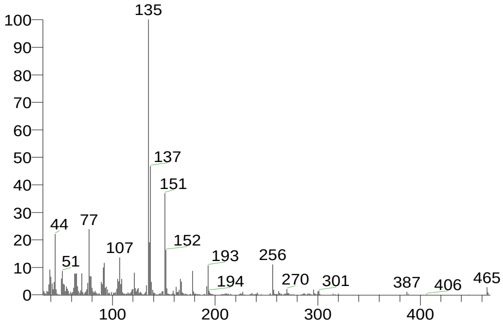

Formula C26H27NO7, MW 465, CAS# NA, Entry# 599252

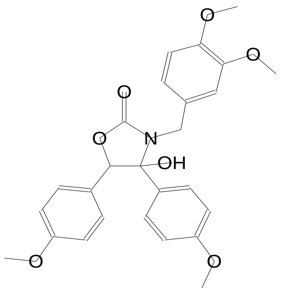

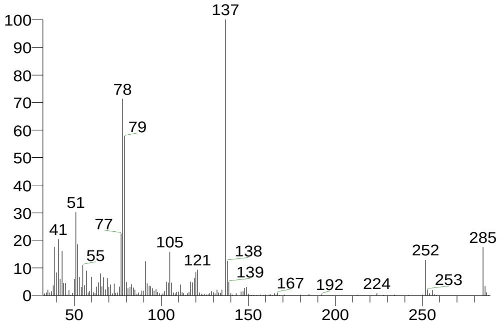

N-(2,5-Dimethyl-phenyl)-2-(pyridin-2-ylamino)-2-thioxo-acetamide
Formula C15H15N3OS, MW 285, CAS# NA, Entry# 340953
N-(2,5-Dimethylphenyl)-2-(2-pyridinylamino)-2-thioxoacetamide

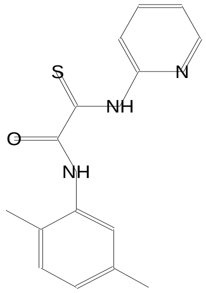

RT	Probability	Compound Name	SI	RSI	Cas #	Area %	Area
52.74	8.16	Benzoic acid, 3,4,5-trihydroxy-, propyl ester (CAS)	493	621	121-79-9	3.07	115071.08
52.74	6.42	2(3H)-OXAZOLONE, 3-[(3,4-DIMETHOXYPHENYL)METHYL]DIHYDRO-4-HYDROXY-4,5-BIS(4-METHOXYPHENYL)-	487	570	NA	3.07	115071.08
52.74	4.78	N-(2,5-Dimethyl-phenyl)-2-(pyridin-2-ylamino)-2-thioxo-acetamide	479	638	NA	3.07	115071.08

There is no signature data to report.

ESOGU-ARUM

Hit Spectrum | Compound Structure

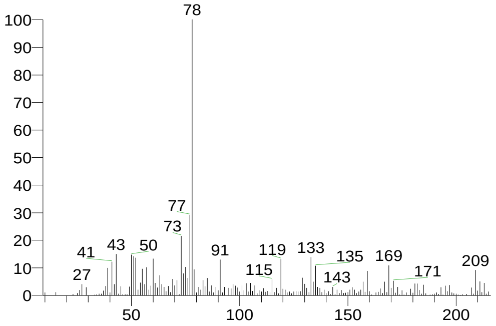

Benzoic acid, 3,4,5-trihydroxy-, propyl ester (CAS)
Formula C10H12O5, MW 212, CAS# 121-79-9, Entry# 168871
Nipa 49

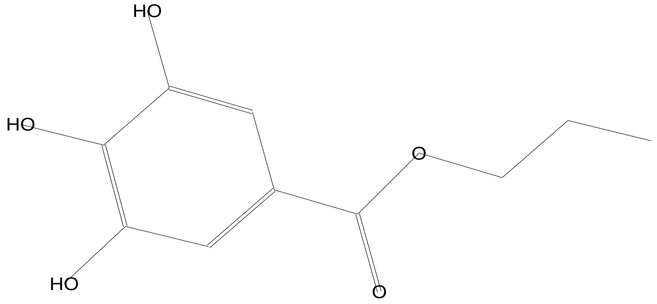

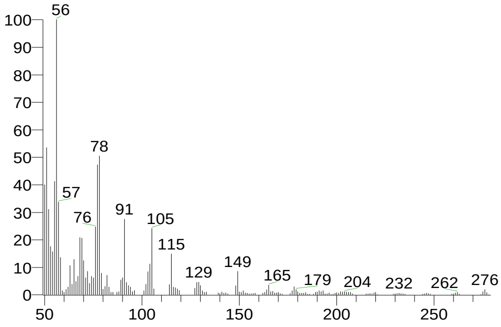

DIMETHYLDIPHENYLTETHYLIDYLPYRROLIDINE
Formula C20H23N, MW 277, CAS# NA, Entry# 323094

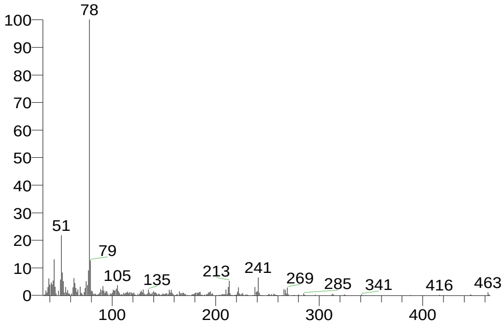

Formula C22H17N5O7, MW 463, CAS# NA, Entry# 597950

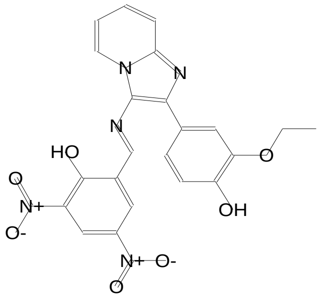

RT	Probability	Compound Name	SI	RSI	Cas #	Area %	Area
53.17	8.52	Benzoic acid, 3,4,5-trihydroxy-, propyl ester (CAS)	498	618	121-79-9	6.41	240147.20
53.17	5.84	DIMETHYLDIPHENYLTETHYLIDYLPYRROLIDINE	487	661	NA	6.41	240147.20
53.17	4.93	2-({[2-(3-ETHOXY-4-HYDROXYPHENYL)IMIDAZO[1,2-A]PYRIDIN-3-YL]IMINO}METHYL)-4,6-DINITROPHENOL	483	568	NA	6.41	240147.20

There is no signature data to report.

ESOGU-ARUM

Hit Spectrum	Compound Structure

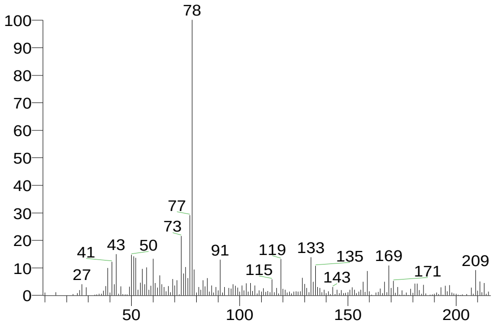

Benzoic acid, 3,4,5-trihydroxy-, propyl ester (CAS)
Formula C10H12O5, MW 212, CAS# 121-79-9, Entry# 168871
Nipa 49

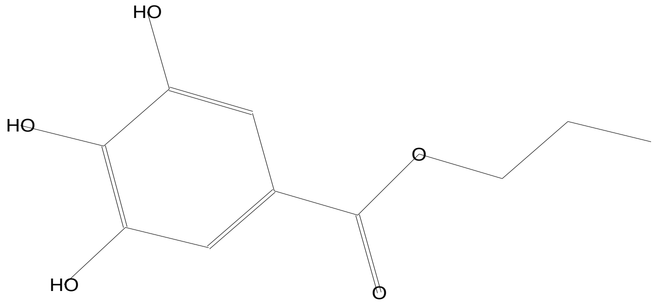

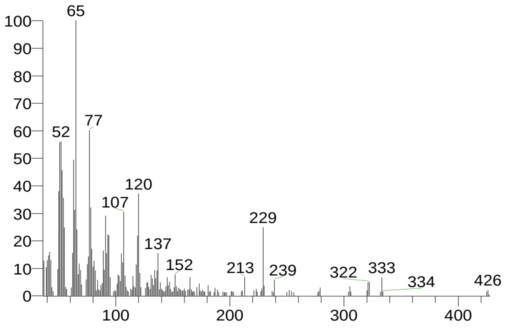

7,12-Dihydro-6,7-bis(4-hydroxyphenyl)-6H-[1,2,4]triazolo[1',5':1,2]pyrimido[5,4-c]chromen-2-ol
Formula C24H18N4O4, MW 426, CAS# NA, Entry# 569540
7,12-DIHYDRO-6,7-BIS(4-HYDROXYPHENYL)-6H-[1,2,4]TRIAZOLO[1',5':1,

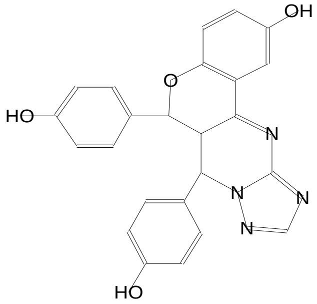

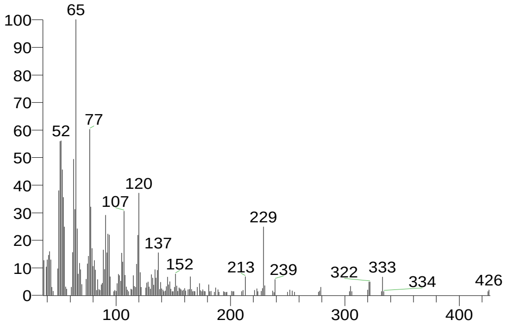

7,12-Dihydro-6,7-bis(4-hydroxyphenyl)-6H-[1,2,4]triazolo[1',5':1,2]pyrimido[5,4-c]chromen-2-ol
Formula C24H18N4O4, MW 426, CAS# NA, Entry# 27977

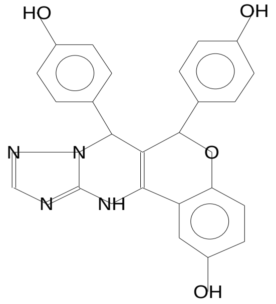

RT	Probability	Compound Name	SI	RSI	Cas #	Area %	Area
53.94	18.51	Benzoic acid, 3,4,5-trihydroxy-, propyl ester (CAS)	517	661	121-79-9	5.98	224106.97
53.94	4.29	7,12-Dihydro-6,7-bis(4-hydroxyphenyl)-6H-[1,2,4]triazolo[1',5':1,2]pyrimido[5,4-c]chromen-2-ol	477	627	NA	5.98	224106.97
53.94	4.29	7,12-Dihydro-6,7-bis(4-hydroxyphenyl)-6H-[1,2,4]triazolo[1',5':1,2]pyrimido[5,4-c]chromen-2-ol	477	626	NA	5.98	224106.97

There is no signature data to report.
