# Supplementary material for: Antioxidant, Redox, and Immunomodulatory Effects of Hypericum perforatum in the Galleria mellonella: A 3R-Oriented Invertebrate Model
Source: Biomedicines. 2026 Jun 7;14(6):1297. doi: 10.3390/biomedicines14061297 (PMC13296390; doi:10.3390/biomedicines14061297)
Supplement: Supplementary file 1 [file biomedicines-14-01297-s001.zip › Supplementary_Material S2.pdf]

Supplementary Material 2 (Statistical Analyses)

This supplementary file provides additional statistical analyses of total protein (TP), catalase (CAT), superoxide dismutase (SOD), and malondialdehyde (MDA) levels measured in *Galleria mellonella* hemolymph following *Hypericum perforatum* administration. Table numbering continues sequentially from the previously submitted Supplementary Table S1. In this context, the data is organized under the following sections: total protein levels are presented under Supplementary Table S1. Statistical analysis of total protein (TP) levels, catalase activity data under Supplementary Table S2. Statistical analysis of catalase (CAT) activity, superoxide dismutase activity results under Supplementary Table S3. Statistical analysis of superoxide dismutase (SOD) activity, and malondialdehyde levels under Supplementary Table S4. Statistical analysis of malondialdehyde (MDA) levels. Regarding immune responses, the parametric and non-parametric test results for each 4h encapsulation variable are summarized under Supplementary Table S5. Summary of parametric and nonparametric test results for each 4h encapsulation variable, while the non-parametric test results for each 24h encapsulation variable are provided under Supplementary Table S6. Summary of nonparametric test results for each 24h encapsulation variable. The statistical analyses of melanization responses, including descriptive statistics (mean ± SD) after asin$\left(\sqrt{percantage}\right)$transformation, are detailed under Supplementary Table S7. Statistical Analysis of Melanization Responses at 4 H Descriptive (mean ± SD) after asin$\left(\sqrt{percantage}\right)$transformation and Supplementary Table S8. Statistical Analysis of Melanization Responses at 24 H Descriptive statistics (mean ± SD) after asin$\left(\sqrt{percantage}\right)$ transformation, respectively. Finally, the statistical evaluations of total hemocyte count results are presented under Supplementary Table S9. Statistical Analysis THC results**.**

Supplementary Table S1. Statistical analysis of total protein (TP) levels

Source of Variation	df	SS	MS	F	p-value
Between Groups	5	0.1210	0.0242	4.764	0.0005
Within Groups	90	0.4568	0.0051	-	-
Total	95	0.5778	-	-	-

Shapiro–Wilk test indicated deviation from normality in DW (p = 0.008) and HP-2 (p < 0.001) groups. Tukey HSD revealed a significant difference between HP-2 and Control (p = 0.036).

Supplementary Table S2. Statistical analysis of catalase (CAT) activity

Source of Variation	df	SS	MS	F	p-value
Between Groups	5	2.151×10^{-5}	4.302×10^{-6}	0.254	0.939
Within Groups	90	1.524×10^{-3}	1.693×10^{-5}	-	-
Total	95	1.546×10^{-3}	-	-	-

All groups met normality assumptions (Shapiro–Wilk, $p > 0.05$). No significant differences were detected; post-hoc analysis was not performed.

Supplementary Table S3. Statistical analysis of superoxide dismutase (SOD) activity

Source of Variation	df	SS	MS	F	p-value
Between Groups	5	0.000033	0.0000066	0.491	0.782
Within Groups	90	0.001210	0.0000134	-	-
Total	95	0.001243	-	-	-

Shapiro–Wilk test confirmed normal distribution in all groups ($p > 0.05$). Levene's test indicated homogeneity of variances ($F = 1.876$, $p = 0.106$). No significant pairwise differences were observed.

Supplementary Table S4. Statistical analysis of malondialdehyde (MDA) levels

Source of Variation	df	SS	MS	F	p-value
Between Groups	5	0.2481	0.04962	1.333	0.258
Within Groups	90	3.347	0.03719	-	-
Total	95	3.595	-	-	-

All groups were normally distributed (Shapiro–Wilk, $p > 0.05$). Levene's test confirmed homogeneity of variances ($F = 0.874$, $p = 0.503$). No significant pairwise differences were detected.

Supplementary Table S5. Summary of parametric and non parametric test results for each 4h encapsulation variable.

Variable	ANOVA p	Kruskal-Wallis p	η^2 (effect size)
None	0.0883	0.2038	0.099 (moderate)
Weak	0.0025	0.00596	0.182 (large)
Strong	0.000285	0.00250	0.226 (large)

Post-hoc comparisons (Tukey HSD)

- **Weak encapsulation:** The HP-3 group showed significantly higher weak encapsulation values than the DW and HP-2 groups ($p < 0.05$ for both).
- **Strong encapsulation:** The HP-2 group showed significantly higher strong encapsulation values than the HP-3 and HP-4 groups ($p < 0.01$ for both).

No other pairwise differences reached statistical significance.

Note on statistical approach

Because normality and homogeneity of variance assumptions were partially violated (notably for None and Strong), both parametric (ANOVA) and non-parametric (Kruskal-Wallis) tests were performed. The consistent results across both methods support the reliability of the findings.

Reporting-ready statistical summary

Kruskal-Wallis tests revealed significant differences among groups for Weak ($\chi^2 = 16.33$, df = 5, p = 0.006) and Strong ($\chi^2 = 18.39$, df = 5, p = 0.002) encapsulation, but not for None (p = 0.204). Effect sizes (η^2) were 0.18 and 0.23, respectively, indicating large effects. Tukey post-hoc comparisons showed that the HP-3 group had significantly higher Weak values than DW and HP-2, and the HP-2 group had significantly higher Strong values than HP-3 and HP-4.

Supplementary Table S6. Summary of non parametric test results for each 24h encapsulation variable.

Variable	Kruskal-Wallis χ^2	df	p	ε^2 (effect size)
None	8.86	5	0.115	0.05 (small)

Variable	Kruskal-Wallis χ^2	df	p	ε^2 (effect size)
Weak	43.94	5	<0.001	0.44 (large)
Strong	41.44	5	<0.001	0.41 (large)

Post-hoc comparisons (Dunn test with Bonferroni correction)

Weak encapsulation:
Significant pairwise differences ($p < 0.05$) were as follows:

Comparison	Z value	Adjusted p	Direction
HP-1 vs HP-3	-3.70	0.0032	HP-3 > HP-1
DW vs HP-4	-4.47	0.0001	HP-4 > DW
HP-1 vs HP-4	-5.70	<0.0001	HP-4 > HP-1
HP-2 vs HP-4	-4.59	<0.0001	HP-4 > HP-2
HP-4 vs Untreated	4.20	0.0004	HP-4 > Untreated

No other pairwise comparisons reached statistical significance. In particular, the difference between HP-3 and HP-4 was not significant.

Strong encapsulation:
Significant pairwise differences ($p < 0.05$) were as follows:

Comparison	Z value	Adjusted p	Direction
HP-1 vs HP-3	3.70	0.0033	HP-1 > HP-3
DW vs HP-4	3.82	0.0020	DW > HP-4
HP-1 vs HP-4	5.91	<0.0001	HP-1 > HP-4
HP-2 vs HP-4	4.55	<0.0001	HP-2 > HP-4
HP-4 vs Untreated	-3.23	0.0189	Untreated > HP-4

No other pairwise comparisons were statistically significant.

Reporting-ready statistical summary

Kruskal-Wallis tests showed no significant differences among groups for None encapsulation ($\chi^2 = 8.86$, df = 5, p = 0.115; $\varepsilon^2 = 0.05$). In contrast, highly significant differences were observed for Weak ($\chi^2 = 43.94$, df = 5, p < 0.001; $\varepsilon^2 = 0.44$) and Strong ($\chi^2 = 41.44$, df = 5, p < 0.001; $\varepsilon^2 = 0.41$). Dunn post-hoc tests with Bonferroni correction revealed that HP-4 had significantly higher Weak values than Untreated, DW, HP-1, and HP-2 (all p < 0.001), and HP-3 had higher Weak values than HP-1 (p = 0.003). For Strong encapsulation, HP-4 had significantly lower values than DW, HP-1, HP-2, and Untreated (all p < 0.02), and HP-1 had higher Strong values than HP-3 (p = 0.003).

Supplementary Table S7. Statistical Analysis of Melanization Responses at 4 H Descriptive statistics (mean ± SD) after asin(sqrt(percentage)) transformation.

Group	None (n=16)	Melanized (n=16)
Untreated	0.2991 ± 0.1871	0.8278 ± 0.2716
DW	0.2845 ± 0.1112	0.8176 ± 0.1577
HP-1	0.3018 ± 0.1875	0.8207 ± 0.2637
HP-2	0.1889 ± 0.1128	0.9741 ± 0.1891
HP-3	0.3514 ± 0.2364	0.7972 ± 0.3740
HP-4	0.3322 ± 0.2353	0.8068 ± 0.3284

Assumption checks (Levene and Shapiro-Wilk tests).

Variable	Levene test (p)	Homogeneous variance?	Shapiro-Wilk (p)	Normality assumption
None	0.2248	Yes	0.0028	Violated
Melanized	0.3444	Yes	0.0125	Violated

ANOVA results.

Variable	Sum of squares (group)	df	Mean square	F value	p value	Significant?
None	0.2557	5	0.05114	1.486	0.202	No
Melanized	0.3510	5	0.07017	0.932	0.464	No

Kruskal-Wallis test results (non-parametric).

Variable	χ^2	df	p value	Significant?
None	7.7325	5	0.1716	No
Melanized	7.7325	5	0.1716	No

Note: The identical χ^2 and p values for both variables reflect the ranking structure of the data and do not affect the conclusion of no significant difference.

One-way ANOVA revealed no significant differences among the six groups (Untreated, DW, HP-1, HP-2, HP-3, HP-4) for None ($F(5,90) = 1.486$, $p = 0.202$) or Melanized ($F(5,90) = 0.932$, $p = 0.464$). Although normality was violated (Shapiro-Wilk $p < 0.05$), variances were homogeneous (Levene $p > 0.05$), and ANOVA is considered robust under these conditions. Kruskal-Wallis tests confirmed the lack of significant differences for None ($\chi^2 = 7.73$, df = 5, $p = 0.172$) and Melanized ($\chi^2 = 7.73$, df = 5, $p = 0.172$). Because no overall significance was found, post-hoc comparisons were not performed.

Supplementary Table S8. Statistical Analysis of Melanization Responses at 24 H Descriptive statistics (mean ± SD) after asin(sqrt(percentage)) transformation.

1. None Melanized

Assumption checks:

- Levene test for homogeneity of variance: p = 4.09e-06 (violated)
- Shapiro-Wilk test for normality of residuals: p = 5.20e-05 (violated)

Due to violated assumptions, a non-parametric Kruskal-Wallis test was used.

Kruskal-Wallis test:
$\chi^2 = 19.11$, df = 5, p = 0.00183 → Significant difference among groups.

Effect size (ε^2, corrected): $\varepsilon^2 = 0.1568$ (moderate-to-large)

Dunn post-hoc test (Holm adjustment):
Significant pairwise differences (p.adj < 0.05):

Comparison	p.adj	Direction
HP-1 – Untreated	0.0052	HP-1 < Untreated
HP-4 – Untreated	0.0011	HP-4 < Untreated

No other pairwise comparisons were significant.

Descriptive statistics (None, asin-transformed):

Group	Mean ± SD	Median	n
Untreated	0.3634 ± 0.2448	0.3972	16
DW	0.1059 ± 0.0717	0.0973	16
HP-1	0.0809 ± 0.0771	0.0871	16
HP-2	0.1222 ± 0.1112	0.0982	16
HP-3	0.1147 ± 0.0823	0.1343	16
HP-4	0.0683 ± 0.0683	0.0558	16

---

2. Melanized Variable

Assumption checks:

- Levene test for homogeneity of variance: p = 0.485 (satisfied)
- Shapiro-Wilk test for normality of residuals: p = 0.00014 (violated)

ANOVA was used because it is robust to moderate deviations from normality when variances are homogeneous.

One-way ANOVA:
$F(5, 90) = 5.647$, $p = 0.000142$ → Significant difference among groups.

Effect size (η^2): $\eta^2 = 0.2388$ (large)

Tukey HSD post-hoc test:
Significant pairwise differences (p.adj < 0.05):

Comparison	Mean difference	p.adj	Direction
DW – Untreated	0.3568	0.0088	DW > Untreated
HP-1 – Untreated	0.4418	0.0005	HP-1 > Untreated
HP-2 – Untreated	0.3405	0.0144	HP-2 > Untreated
HP-3 – Untreated	0.3542	0.0095	HP-3 > Untreated
HP-4 – Untreated	0.4808	0.00012	HP-4 > Untreated

No other pairwise comparisons (e.g., DW vs HP-1, HP-2 vs HP-3) were significant. Thus, all treatment groups (DW, HP-1, HP-2, HP-3, HP-4) showed significantly higher Melanized values than the Untreated group, but did not differ among themselves.

Descriptive statistics (Melanized, asin-transformed):

Group	Mean ± SD	Median	n
Untreated	0.8032 ± 0.4220	0.6613	16
DW	1.1601 ± 0.2256	1.1265	16
HP-1	1.2450 ± 0.2513	1.1507	16
HP-2	1.1438 ± 0.2713	1.1245	16
HP-3	1.1574 ± 0.2615	1.0476	16
HP-4	1.2840 ± 0.2465	1.2355	16

For the None variable, a Kruskal-Wallis test revealed significant differences among groups ($\chi^2 = 19.11$, df = 5, $p = 0.00183$; $\varepsilon^2 = 0.1568$). Dunn post-hoc tests (Holm-adjusted) showed that HP-1 and HP-4 had significantly lower None values than the Untreated group ($p = 0.0052$ and $p = 0.0011$, respectively). No other pairwise differences were significant.

For the Melanized variable, a one-way ANOVA demonstrated significant differences among groups ($F(5,90) = 5.647$, $p = 0.000142$; $\eta^2 = 0.2388$). Tukey HSD post-hoc tests indicated

that all treatment groups (DW, HP-1, HP-2, HP-3, HP-4) had significantly higher Melanized values than the Untreated group (all $p < 0.05$), with no significant differences among the treatment groups themselves.

Supplementary Table S9. Statistical Analysis THC results.

One-way Analysis of Variance (ANOVA) for treatment effects

Source of Variation	df	SS	MS	F	p-value
**Between Groups**	5	35660.92	7132.18	5.36	0.00023
**Within Groups**	90	119730.25	1330.34	-	-
**Total**	95	155391.17	-	-	-

Descriptive statistics of experimental groups (per group)

Group	Mean ()	SD	SEM
Untreated	208.19	15.56	3.89
Distilled Water	216.81	43.24	10.81
HP-1	244.75	23.75	5.94
HP-2	263.75	44.25	11.06
HP-3	221.75	48.96	12.24
HP-4	208.56	30.91	7.73

Statistical Notes and Assumptions

- **Normality & Homogeneity:** Data distribution was assessed using the Shapiro–Wilk test, confirming normality across all groups ($p>0.05$). Levene's test indicated homogeneity of variances ($p=0.068$).
- **Effect Size:** The calculated Eta-squared ($\eta^2=0.23$) indicates a large effect size, suggesting that treatment type explains approximately **22.95%** of the total variance.
- **Post Hoc Analysis:** Tukey HSD test revealed that the **HP-2** group was significantly higher than the Untreated ($p=0.001$), HP-4 ($p=0.001$), Distilled Water ($p=0.010$), and HP-3 ($p=0.027$) groups. Additionally, **HP-1**showed a significant increase

compared to the Untreated group ($p=0.049$). No other significant differences were observed between the remaining groups ($p>0.05$).
